# A model-based framework for chronic hepatitis C prevalence estimation

**DOI:** 10.1371/journal.pone.0225366

**Published:** 2019-11-21

**Authors:** Abdullah Hamadeh, Zeny Feng, Murray Krahn, William W. L. Wong

**Affiliations:** 1 School of Pharmacy, University of Waterloo, Kitchener, ON, Canada; 2 Department of Mathematics and Statistics, University of Guelph, Guelph, ON, Canada; 3 Toronto Health Economics and Technology Assessment Collaborative, University Health Network, Toronto General Hospital, Toronto, ON, Canada; 4 Toronto General Research Institute, Toronto, ON, Canada; Centre de Recherche en Cancerologie de Lyon, FRANCE

## Abstract

Chronic hepatitis C (CHC) continues to be a highly burdensome disease worldwide. The often-asymptomatic nature of early-stage CHC means that the disease often remains undiagnosed, leaving its prevalence highly uncertain. This generates significant uncertainty in the planning of hepatitis C eradication programs to meet WHO targets. The aim of this work is to establish a mathematical framework for the estimation of a geographic locale’s CHC prevalence and the proportion of its CHC population that remains undiagnosed. A Bayesian MCMC approach is taken to infer these populations from the observed occurrence of CHC-related events using a recently published natural history model of the disease. Using the Canadian context as a specific example, this study estimates that in 2013, the CHC prevalence rate in Canada was 0.63% (95% CI: 0.53% - 0.72%), with 27.1% (95% CI: 19.3% - 36.1%) of the infected population undiagnosed.

## Introduction

Chronic hepatitis C (CHC) is a progressive and infectious disease that can remain asymptomatic for decades before ultimately causing liver damage, liver failure and liver cancer. Hepatitis C-related illness annually claims in excess of 399,000 lives worldwide [1]. The primary modes of transmission of the hepatitis C virus (HCV) include injection drug use and blood transfusion [1].

New and highly effective therapies for HCV chronic infections, in the form of direct acting antivirals (DAA), present the opportunity to meet the WHO hepatitis C eradication target of 2030. However, the planning of eradication strategies is highly sensitive to estimates of the CHC prevalence and diagnosis rates [2]. Because CHC often remains asymptomatic until its late-stage, there is considerable uncertainty in current prevalence estimates. Furthermore, CHC treatment with DAAs remains expensive, making estimates of budget impact from the public funding of DAAs also highly uncertain and sensitive to estimates of disease prevalence.

In this paper, we present a model-based framework for the inference of CHC-infected populations, using the Canadian context as a specific example. The case of Canada is of particular interest for two reasons: the uncertainty in CHC prevalence is compounded by the absence of a population-level hepatitis C screening program. In addition, there is limited public data available from which to infer the size of the HCV-infected population.

In a 2007 Public Health Agency of Canada study, an actuarial method was used to estimate hepatitis C prevalence in Canada [3]. The population was divided into subgroups according to their risk of having or contracting hepatitis C. Rates of prevalence and incidence were estimated for each subpopulation, and these were then used in a state transition model to obtain projections of the total number of individuals with hepatitis C-induced liver diseases.

Thein et al. [4] reported the results of a systematic review of annual probabilities of fibrosis progression. A study of the 2011 prevalence rate of hepatitis C in Canada [5] incorporated the rates in [4] into a Markov model of disease progression and used a backcalculation method to obtain rate estimates. Alongside the backcalculation method, an actuarial method was also used, stratifying the Canadian population according to the risk of infection and prevalence. While the backcalculation estimated the 2011 prevalence of chronic hepatitis C to be 0.64% of the Canadian population, the actuarial approach yielded a higher rate of 0.71%. The backcalculation methodology and model assumptions used in [5] were not disclosed.

In 2015, a new Markov model of the natural history of hepatitis C in Canada was developed as part of economic evaluations of hepatitis C-related screening and treatment [6]. This model has been used by various agencies in Canada for decision making in hepatitis C-related policies on screening and treatment [2]. This model divided the progression of hepatitis C into two broad stages: pre-advanced liver disease and advanced liver disease. The population of individuals in the pre-advanced stage of hepatitis C was modeled as progressing through the liver fibrosis stages of the disease at rates that were obtained from [4]. The model additionally included the diagnosis of patients at the various fibrosis stages as well as their subsequent treatment and response to treatment. Patients who progressed to the last fibrosis stage were modeled as being at risk of progressing to advanced liver disease: either hepatocellular carcinoma or decompensated cirrhosis. From these latter stages, the model captured the progression of patients through advanced liver disease, including receipt of liver transplants, liver-related deaths and non-liver related deaths.

In this paper, we adopt a Bayesian Markov Chain Monte Carlo approach, based on the Metropolis-Hastings algorithm [7], to estimate the Canadian CHC prevalence and undiagnosed CHC population. We derive a comprehensive mathematical framework for the back-calculation using the model reported in [6]. The estimates are obtained by calibrating the model using publicly available hepatocellular carcinoma (HCC) diagnosis data from Statistics Canada [8] and HCV diagnosis data from the Public Health Agency of Canada [9], together with estimates of liver fibrosis distributions at diagnosis from [6].

This paper is organized as follows: First, a qualitative description of the hepatitis C natural history model of [6] is provided followed by a discussion of its key parameters and its mathematical formulation. An overview of the MCMC algorithm used in this study is then provided, followed by the modeling results and a discussion of key conclusions.

## Methods

The aim of this work is to establish a mathematical framework for the estimation of CHC populations of interest. For the illustrative case of Canada, we focus on the estimation of the historical prevalence of CHC and the proportion of the CHC population that is undiagnosed for each of three birth cohorts:

- individuals born before 1945,
- individuals born between 1945–1964,
- individuals born after 1964.

We first give an overview of a state transition model, introduced in [6], that describes the natural history of hepatitis C within a single, generic, birth cohort. Each state of the model corresponds to the number of individuals, within the cohort, that populate a particular CHC stage and that fall under a particular patient status. We next give an overview of the model parameters and introduce a mathematical formulation of the model dynamics that describe the transition of patients from one state to another between an initial year 0 and a final year *T*. Using this formulation, we demonstrate how the model is able to simulate the number of individuals in each state of the disease between years 0 and *T*, inclusive, for a given set of input parameters. This input parameter set includes state transition probabilities, the states of the model in year 0 and the number of new infections in the *T* subsequent years. We then describe how the simulation results can be used to estimate the cohort’s expected CHC prevalence, undiagnosed CHC population, as well as the expected number of HCC and HCV diagnoses from within the cohort in the years 0 to *T*.

Arriving at estimates in this way, however, relies on knowledge of the input parameter set, which is not readily available from the literature. Therefore, we then present a Bayesian calibration method, based on the Metropolis-Hastings algorithm, through which these unknown quantities are estimated from recorded HCC and HCV diagnosis data and from statistics on the stages of liver fibrosis that are observed upon HCV diagnosis.

### The hepatitis C natural history model

We assume that for the entirety of any given calendar year, a CHC patient’s disease state can be categorized into only one of several stages of the disease. The actual number of individuals in each disease state is not known. Such latent variables will, over the course of time, change as patients progress from one disease state to the next. On the other hand, there are observables arising from HCV-related events, such as the diagnosis of a patient with HCV infection or HCC. Such observables are consequences of HCV infection and so depend directly on the latent variables. As a result, these observables provide important information for estimating the unknown latent variables.

**Fig 1:**
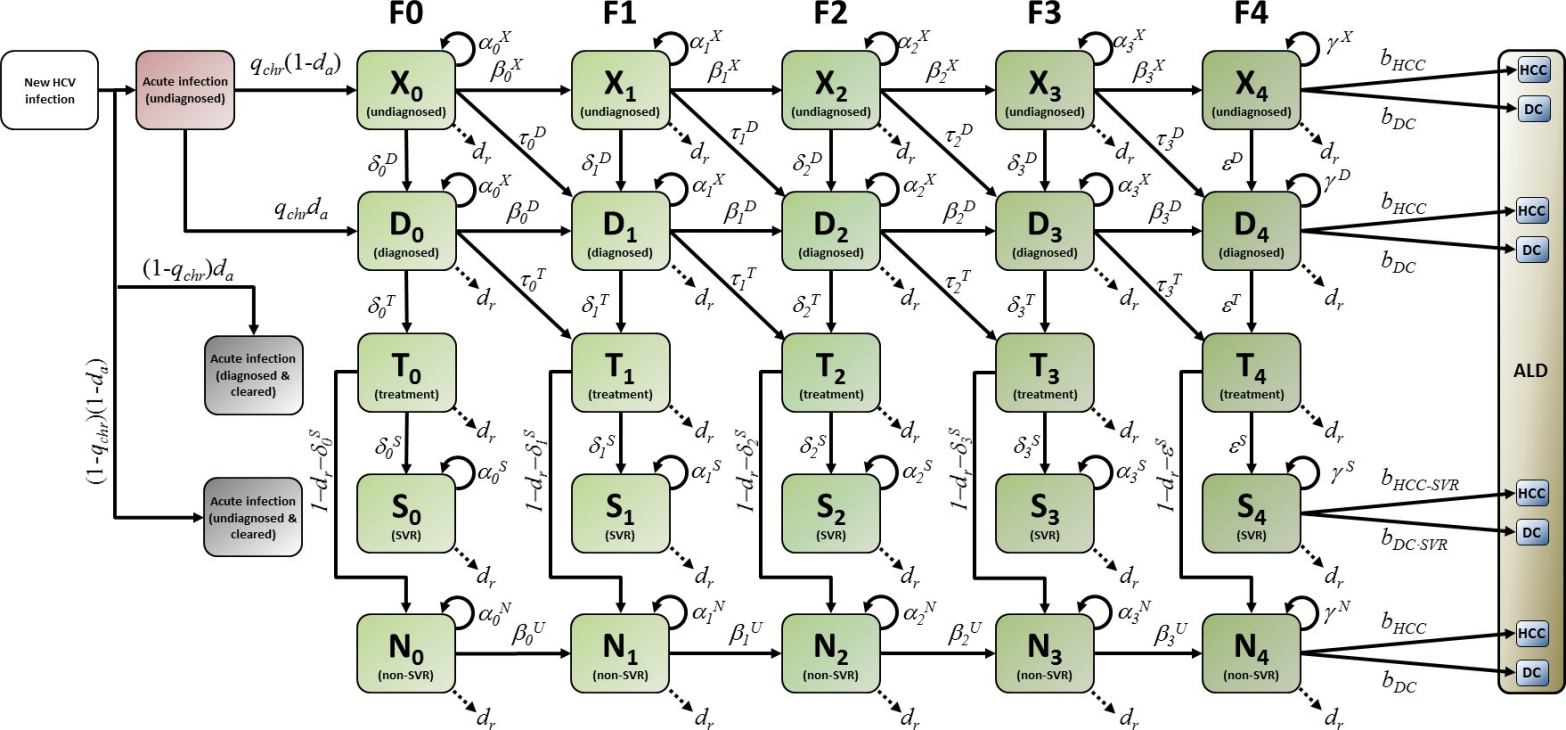
State transition model of early hepatitis C natural history. ALD: Advanced liver disease. SVR: Sustained virologic response.

**Fig 2:**
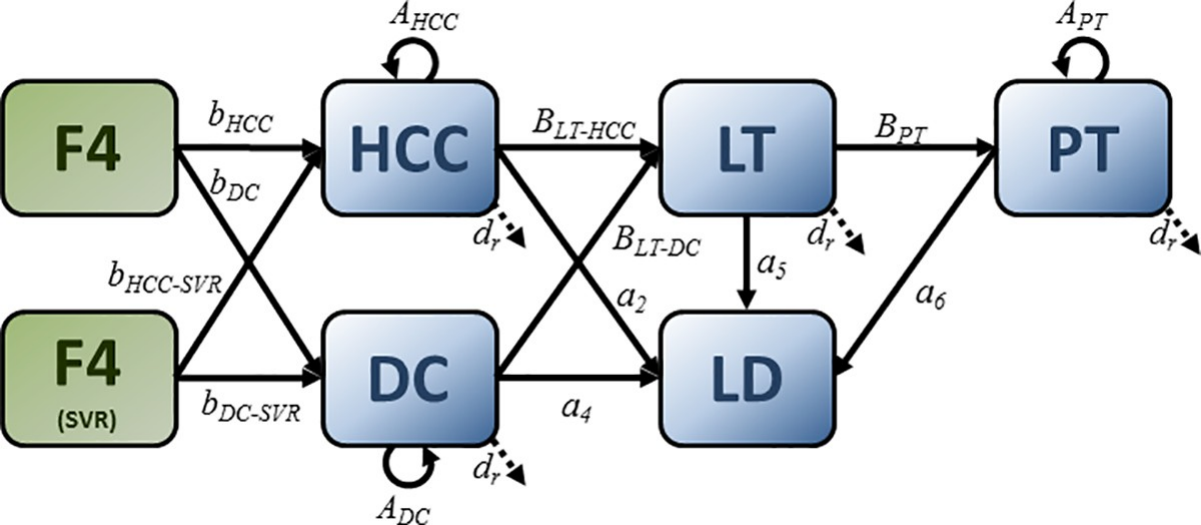
Advanced liver disease state transition model. **Transition probabilities are derived in S1 Appendix. HCC**: hepatocellular carcinoma. **DC**: decompensated cirrhosis. **LT**: liver transplant. **PT**: post-liver transplant. **LD**: liver-related death. **SVR**: Sustained virologic response.

The state transition model illustrated in Fig 1 is assumed to describe the movement of hepatitis C patients, within one birth cohort, between the various latent states of hepatitis C infection that precede advanced liver disease. A state transition model of progression between the advanced liver disease stages is illustrated in Fig 2. The transition probabilities emanating from each state in Fig 1 and Fig 2 sum to one, and are derived in S1 Appendix as functions of annual probabilities of fibrosis progression, CHC diagnosis, background death, treatment and SVR.

It is assumed that in the year *t* there are *u*(*t*) new HCV infections. Newly infected patients are assumed to have a chance of clearing the virus in the acute stage of infection [10]. If the virus is not cleared, the disease becomes chronic and the patient risks developing increasingly advanced levels of liver fibrosis. The model assumes that HCV infection may be diagnosed in either the acute or the chronic stages.

The degree of liver fibrosis is divided into five stages, denoted F0, F1, F2, F3 and F4 [6]. In Fig 1, individuals who have CHC but are undiagnosed are grouped into latent states X_0_, X_1_, X_2_, X_3_, X_4_, where the subscripts represent the five fibrosis levels F0-F4. Individuals who have CHC and are diagnosed but have not yet received treatment are similarly grouped into latent states D_0_, D_1_, D_2_, D_3_, D_4_. Individuals who have CHC and who are receiving treatment are grouped into latent states T_0_, T_1_, T_2_, T_3_, T_4_. A patient treated for CHC in one year may, the following year, show SVR. Individuals who show SVR following treatment are grouped into latent states S_0_, S_1_, S_2_, S_3_, S_4_. Once in these SVR states, it is assumed that patients are no longer at risk of progressing in liver fibrosis severity. Individuals who do not show SVR following treatment are grouped into latent states N_0_, N_1_, N_2_, N_3_, N_4_ and their liver fibrosis is assumed to progress at a rate equal to that of the untreated population. CHC patients who reach the cirrhosis stage F4 (latent states X_4_, D_4_, T_4_, S_4_, N_4_) become at risk of developing more advanced liver disease. Advanced liver disease (ALD) states are categorized into the following states:

- DC: patients with decompensated cirrhosis,
- HCC: patients with hepatocellular carcinoma,
- LT: patients receiving liver transplants,
- PT: patients who have survived more than one year after their liver transplantation.

The model assumes that individuals in these advanced liver disease states may transition to a liver-related death state (LD), with a probability greater than the background all-cause mortality.

### Parameters of the natural history model

#### Viral genotype

The hepatitis C virus has six major genotypes [11], which we denote *v* = 1,⋯,6. The distribution of the prevalence of the different genotypes in Canada is reported in [6]. The genotype affects the treatment success rate and, consequently, the treatment adoption rate.

#### Model transition probabilities

We denote by *q*_*chr*_ the annual probability of a newly infected individual transitioning from an acute to a chronic infection. S1 Table gives the mean value of *q*_*chr*_ as well as its range (from [10]), over which we assume it is uniformly distributed.

For individuals with CHC who do not show a sustained virologic response, we let *q*_*i*,*i*+1_ be the annual probability of progression between any two consecutive fibrosis stages F_*i*_ and F_*i*+1_. The annual probability of fibrosis level progression varies depending on viral genotype, and we assume each parameter *q*_*i*,*i*+1_ to be uniformly distributed within a range of values that captures this variation. These ranges, which we derive from the systematic review of progression statistics for CHC patients in [4], are given in S1 Table. For patients showing a sustained virologic response, the probability of fibrosis stage progression is assumed to be zero. S1 Table additionally gives means and ranges of the annual probabilities of progression between the stages of advanced liver disease, denoted *a*_1_,⋯,*a*_6_, which are assumed to be uniformly distributed.

We assume that the symptoms of hepatocellular carcinoma and decompensated cirrhosis are severe enough that an individual’s progression to, and subsequent diagnosis with, either HCC or DC occur in the same calendar year. We additionally assume that patients who were previously undiagnosed with CHC and who progress to advanced liver disease are additionally diagnosed with CHC upon their diagnosis with the ALD. The annual probability of progression to decompensated cirrhosis is denoted by *d*_*DC*_ for non-SVR individuals and by *d*_*DC*−*SVR*_ for patients who show SVR. The parameters *d*_*DC*_ and *d*_*DC*−*SVR*_ are assumed to be uniformly distributed, with their means and ranges, reported in [12], given in S1 Table. The corresponding probabilities of progression to hepatocellular carcinoma, respectively denoted *d*_*HCC*_ and *d*_*HCC*−*SVR*_, will be estimated.

We let *d*_*a*_ be the annual acute hepatitis C probability of diagnosis. We also denote by *d*_*i*_ the annual CHC probability of diagnosis for fibrosis stage F_*i*_. These parameters are not available from the literature and will be estimated.

We denote by *t*_*i*_(*v*) the annual probability that a patient who was previously diagnosed with CHC of viral genotype *v* and who is currently at fibrosis stage F_*i*_, will adopt pegylated interferon and ribavirin therapy. The estimated cumulative probabilities of starting treatment over the period 1999–2013 in Canada, per fibrosis stage and genotype were reported in [6]. We converted these cumulative probabilities to annual probabilities of treatment *t*_*i*_(*v*), given in S2 Table.

We let *s*_*i*_(*v*) be the probability that an individual infected with CHC, of genotype *v*, who receives treatment when at fibrosis stage F_i_ will show a sustained virologic response. The probability of a patient not showing a sustained virologic response at that fibrosis stage is then 1−*s*_*i*_(*v*). Wong et al., [6], report the probabilities of SVR by fibrosis stage and viral genotype following pegylated interferon and ribavarin therapy.

We denote the annual probability of background death (i.e. all-cause mortality) for HCV-infected individuals in a year *t* by *d*_*r*_(*t*). For the specific example of Canada [13], the background mortality among CHC patients with respect to the general population varies with age. In particular, with respect to the general population, younger CHC patients are at an increased risk of mortality from activities associated with HCV acquisition [14]. One such activity is injection drug use, for which the standard mortality ratio is 16.4 (95% CI: 9.1–27.1) [3,15,16] and which accounts for 70–80% of new HCV injections in Canada [17]. In contrast, older patients are more likely to die of liver-related causes [14], which are accounted for in the model through the advanced liver disease states. Therefore, as a simplifying assumption, we assume that infected individuals born before 1965 have a background mortality equal to that of the general population.

### Mathematical formulation of natural history model

#### State transition modeling

We denote the *expected number* of individuals in any one of the disease states X_*i*_, D_*i*_, T_*i*_, S_*i*_, N_*i*_, HCC, DC, LT, PT (*i* = 0,1,2,3,4) within a given birth cohort in a year *t* by *X*_*i*_(*t*), *D*_*i*_(*t*), *T*_*i*_(*t*), *S*_*i*_(*t*), *N*_*i*_(*t*), *HCC*(*t*), *DC*(*t*), *LT*(*t*), *PT*(*t*) respectively. We define a state vector *x*(*t*) that consists of the expected number of individuals in each latent state:

$$x(t)=[X{\left(t\right)}^{\prime },D{\left(t\right)}^{\prime },T{\left(t\right)}^{\prime },S{\left(t\right)}^{\prime },N{\left(t\right)}^{\prime },HCC{\left(t\right)}^{\prime },DC{\left(t\right)}^{\prime },LT{\left(t\right)}^{\prime },PT{\left(t\right)}^{\prime }]\prime$$ (1)

where *X*(*t*) = [*X*_0_(*t*) ⋯ *X*_4_(*t*)]′ and likewise for *D*(*t*), *T*(*t*), *S*(*t*), *N*(*t*).

To model changes in *x*(*t*) over time, we use the state transition model described illustrated in Fig 1 and Fig 2. The elements of the vector *x*(*t*+1) can be written in terms of *u*(*t*) (the number of new infections in year *t*) and the elements of the vector *x*(*t*), as follows:

$$X(t+1)=A_{X}(t)X(t)+(1-d_{a})B_{u}(t)u(t)$$ (2A)

$$D(t+1)=A_{D}(t)D(t)+B_{D}(t)X(t)+d_{a}B_{u}(t)u(t)$$ (2B)

$$T(t+1)={A_{T}(t)T(t)+B}_{T}(t)D(t)$$ (2C)

$$S(t+1)=A_{S}(t)S(t)+B_{S}(t)T(t)$$ (2D)

$$N(t+1)=A_{N}(t)N(t)+B_{N}(t)T(t)$$ (2E)

$$HCC(t+1)=A_{HCC}HCC(t)+B_{HCC}(X(t)+D(t)+N(t))+B_{HCC-SVR}S(t)$$ (2F)

$$DC(t+1)=A_{DC}DC(t)+B_{DC}(X(t)+D(t)+N(t))+B_{DC-SVR}S(t)$$ (2G)

$$LT(t+1)=B_{LT-HCC}HCC(t)+B_{LT-DC}DC(t)$$ (2H)

$$PT(t+1)=A_{PT}PT(t)+B_{PT}LT(t)$$ (2I)

Definitions of the terms in (2) are given in S1 Appendix.

In this study, patients who show SVR are assumed to be ‘cured’ of the disease, and therefore do not contribute to the total CHC prevalence from the time they reach SVR. The expected total number of individuals with CHC within a cohort in a year *t*, denoted *P*_*x*_(*t*), is given by

$$P_{x}(t)=\sum_{i=0}^{4}\left((X_{i}(t)+D_{i}(t)+T_{i}(t)+N_{i}(t)\right)+DC(t)+HCC(t)+LT(t)+PT(t)$$ (3)

The prevalence rate *R*_*x*_(*t*) within a given cohort in a year *t* is then given by

$$R_{x}(t)=100\times P_{x}(t)/p_{n}(t)$$ (4)

where *p*_*n*_(*t*) is the cohort’s average population in year *t*. Estimates of *p*_*n*_(*t*) are available online from Statistics Canada [18] and are given in S4 Table.

The expected proportion of the cohort’s CHC population that is undiagnosed, ${\bar{D}}_{x}$, in year *t* is given by the ratio

$${\bar{D}}_{x}(t)=100\times \frac{\sum_{i=0}^{i=4}X_{i}(t)}{P_{x}(t)}$$ (5)

#### Model observables

The model’s expected total number of HCC cases diagnosed in a given cohort in a year *t* (including HCC cases not induced by CHC), is given by

$$y_{HCC}(t)=\frac{1}{c_{HCC}}(d_{HCC}(X_{4}{(t)+D}_{4}{(t)+N}_{4}(t))+d_{HCC_{SVR}}S_{4}(t))$$ (6)

where *c*_*HCC*_ is the proportion of all diagnosed HCC cases that are CHC-induced.

The total number of HCC cases diagnosed each year in Canada and in each province is available from Statistics Canada [8]. By comparing the number of CHC-induced HCC cases diagnosed in British Columbia [19] with the total number of HCC cases diagnosed in that province between 1990 and 2012 (from [8]), we estimated the percentage of all HCC diagnoses that were attributable to chronic hepatitis C, for the three birth cohorts we consider. These estimates are summarized in S3 Table.

Let *y*_*acute*_(*t*) = *d*_*a*_*u*(*t*) be the model’s expected number of acute hepatitis C diagnoses in year *t* and let $y_{F_{i}}(t)$ be the expected number of CHC diagnoses at fibrosis state F_*i*_ in year *t*. The total number of chronic cases diagnosed in year *t*, denoted *y*_*CHC*_(*t*), is then

$$y_{CHC}(t)=\sum_{i=0}^{4}y_{F_{i}}(t)$$ (7)

where

$$y_{F_{i}}(t)={d_{i}(t)X}_{i}(t)\;\mathrm{for}\;i=0,1,2,3,$$ (8A)

$$y_{F4}(t)={d_{4}(t)X}_{4}(t)+d_{HCC}X_{4}(t)+d_{DC}X_{4}(t).$$ (8B)

The model’s expected total number of hepatitis C cases diagnosed in year *t* for a given cohort, denoted *y*_*hepC*_(*t*), is the sum of diagnosed acute cases and chronic cases

$$y_{hepC}(t)=y_{acute}(t)+y_{CHC}(t)$$ (9)

For notational purposes, we define the vector of time-series observables:

$$y(t):=[y_{HCC}(t)\;y_{hepC}(t)]\prime .$$

#### Solutions to the model equations

Eqs (2), (6) and (9) are linear in *u*(*t*) and in the elements of *x*(*t*). The natural history model can therefore be expressed in the concise state-space form:

x(t+1)=A(t)x(t)+B(t)u(t) (10)

y(t)=C(t)x(t)+D(t)u(t) (11)

Where matrices *A*(*t*), *B*(*t*), *C*(*t*), *D*(*t*) are given in S1 Appendix. If the expected disease state vector *x*(*t*) is known at a starting year *t* = 0, and if the expected number of yearly new infections *u*(*t*) is known for years *t* = 0⋯,*T*, we can use (10) to obtain *x*(*t*) for 0<*t*≤*T* as

$$x(t)=(\prod_{i=0}^{t-1}A(i))x_{0}+\sum_{i=0}^{t-2}\left(\prod_{j=i+1}^{t-2}A(j))B(i)u(i\right)+B(t-1)u(t-1)$$ (12)

Together with (11), this yields the expected number of diagnoses for year *t*≥0 as:

$$\begin{array}{l} y(t)=C(t)x(t)+D(t)u(t)\\ =C(t)\left((\prod_{i=0}^{t-1}A(i))x_{0}+\sum_{i=0}^{t-2}\left(\prod_{j=i+1}^{t-2}A(j))B(i)u(i\right)\right)+C(t)B(t-1)u(t-1)+D(t)u(t) \end{array}$$ (13)

For this analysis, observational data on the diagnoses of HCC and HCV were only available for the calendar years 1999–2013. We therefore set the start year of the analysis, *t* = 0, at 1999 and denote by *t* = *T*, with *T* = 14, the last year of the analysis, 2013.

#### Model calibration

Using Eqs (4) and (5), estimates of the prevalence rate *R*_*x*_(*t*) and the undiagnosed proportion ${\bar{D}}_{x}(t)$ can be obtained from estimates of the vector *x*(*t*). The estimate of *x*(*t*) in (12) is a function of many input parameters, only some of which are available from the literature. We organize these literature-derived input parameters of the model into a vector

$$V={\left[{v,q}_{chr},q^{\prime },d_{DC},d_{DC-SVR},a^{\prime },t{\left(v\right)}^{\prime },s{\left(v\right)}^{\prime },d_{r},c_{HCC}\right]}^{\prime }$$ (14)

with *q* = [*q*_01_,*q*_12_,*q*_23_,*q*_34_]′, *a* = [*a*_1_,*a*_2_,*a*_3_,*a*_4_,*a*_5_,*a*_6_]′, *t*(*v*) = [*t*_0_(*v*),*t*_1_(*v*),*t*_2_(*v*),*t*_3_(*v*),*t*_4_(*v*)]′, and *s*(*v*) = [*s*_0_(*v*),*s*_1_(*v*),*s*_2_(*v*),*s*_3_(*v*),*s*_4_(*v*)]′, where *t*_*i*_(*v*) and *s*_*i*_(*v*) are the annual probabilities of treatment adoption and of SVR for fibrosis stage F_*i*_ and viral genotype *v*. As *V* can be treated as a vector of random variables, we denote by *P*(*V*) the joint probability distribution of these parameters.

On the other hand, input parameters in model (12), (13) that are not available from the literature can be grouped into three categories:

### a) Unknown initial year data

From (12), the estimate of *x*(*t*) (0<*t*≤14) is a function of the vector *x*(0), the number of individuals in each state of the model in year *t* = 0. However, since estimates of *x*(0) are not available in the literature, it is a vector that needs to be calibrated. In the calibration, elements of *x*(0) are constrained by the following assumptions:

- Individuals, who in year *t* = 0,
  ○ had previously been diagnosed but had not received treatment, (*D*_*i*_(0)), or,
  ○ were receiving treatment (*T*_*i*_(0)), or
  ○ were non-SVR (*N*_*i*_(0)),
  were assumed to be either in ‘early CHC’ fibrosis stages (respectively denoted *D*_*E*_,*T*_*E*_,*N*_*E*_) or in ‘late CHC’ fibrosis stages (respectively denoted *D*_*L*_,*T*_*L*_,*N*_*L*_).
- Patients in the ‘early CHC’ stage were equally distributed among fibrosis levels F0, F1 and F2, while patients in ‘late CHC’ were equally distributed between fibrosis levels F3 and F4. Formally, we imposed the constraints *D*_0_(0) = *D*_1_(0) = *D*_2_(0) = *D*_*E*_, and *D*_3_(0) = *D*_4_(0) = *D*_*L*_. Analogous constraints were placed on *T*_*i*_(0) and *N*_*i*_(0).
- The number of individuals in an early CHC state was constrained to vary by a factor between 0.5 and 2 with respect to the late CHC state. Formally, we have *D*_*L*_ = *ν*_*D*_*D*_*E*_, *T*_*L*_ = *ν*_*T*_*T*_*E*_, and *N*_*L*_ = *ν*_*N*_*N*_*E*_, where 0.5≤*ν*_*D*_,*ν*_*T*_,*ν*_*N*_≤2.
- Initial states *S*_0_(0)…*S*_3_(0) were not calibrated since the model assumes that no eventual progression in fibrosis or to advanced liver disease is possible from these states, and their values therefore do not impact the number of HCC and hepatitis C diagnoses.
- The ALD initial states *HCC*(0), *DC*(0), *LT*(0) and *PT*(0) were not calibrated since progression to these states only occurs upon or after diagnosis with HCC and hepatitis C (the model observables that are used for calibration).

### b) Unknown yearly new infections

The yearly incidence of new infections *u*(*t*), for *t* = 0,⋯,14 is unknown. We assume that, at the population level, the number of new infections occurring yearly will vary over time in line with trends in activities that carry a high HCV-acquisition risk, such as injection drug use. As a simplifying approximation, we assume that this trend is linear between *t* = 0,⋯,14. We let *u*(0) = *u*_0_ and *u*(14) = *u*_14_ respectively be the number of new infections in years *t* = 0 and *t* = 14. The number of new infections in the intervening years is evaluated through linear interpolation between *u*_0_ and *u*_14_.

### c) Unknown probabilities of diagnosis

We treat as unknown parameters the annual acute hepatitis C probability of diagnosis *d*_*a*_, the annual probability of CHC diagnosis at the F_*i*_ fibrosis stage, *d*_*i*_, the annual probability of progression from F4 to HCC for non-SVR individuals, *d*_*HCC*_, and for SVR individuals, *d*_*HCC*−*SVR*_. We assume that aggregate diagnosis rates are driven by trends in the general awareness of HCV infection and access to testing facilities. Furthermore, for simplicity, we assume that these trends are linear between in years *t* = 0 and *t* = 14. The parameters *d*_*a*_, *d*_*i*_, *d*_*HCC*_, and *d*_*HCC*−*SVR*_ therefore satisfy the following constraints:

- In any given year *t*, the annual probabilities of diagnosis of acute hepatitis C and chronic hepatitis C at fibrosis stage F0 and F1 are equal. That is, *d*_*a*_(*t*) = *d*_0_(*t*) = *d*_1_(*t*)≕*d*_*early*_(*t*). We let $d_{early}(0)={d_{early}}_{0}$ and $d_{early}(14)={d_{early}}_{14}$. The value of *d*_*early*_(*t*) in intervening years is evaluated through linear interpolation between ${d_{early}}_{0}$ and ${d_{early}}_{14}$.
- The annual probabilities of diagnosis of chronic hepatitis C at fibrosis stages F2, F3, F4 are equal. That is *d*_2_(*t*) = *d*_3_(*t*) = *d*_4_(*t*)≕*d*_*late*_(*t*). We let $d_{late}(0)={d_{late}}_{0}$ and $d_{late}(14)={d_{late}}_{14}$. The value of *d*_*late*_(*t*) in intervening years is evaluated through linear interpolation between ${d_{late}}_{0}$ and ${d_{late}}_{14}$.
- The annual probabilities of progression from stage F4 to HCC, *d*_*HCC*_(*t*) is assumed to vary linearly between years *t* = 0 and *t* = 14, with $d_{HCC}(0)={d_{HCC}}_{0}$ and $d_{HCC}(14)={d_{HCC}}_{14}$.
- Following [12], we assume that the annual probability of progression to HCC among F4 patients with SVR is a fifth of that among non-SVR F4 patients.

The ranges of the twenty parameters to be estimated are listed in Table 1. We organize these parameters into a vector *M* and assume that each parameter has a flat uniform prior distribution over the ranges given. Priors for initial states and for the numbers of new infections were based on the previous estimates of hepatitis C incidence and prevalence in [3]. Priors for annual probabilities of diagnoses with HCV infection were based on the model validation study in [6]. Priors for annual probabilities of progression to HCC were based on estimates in [12]. The joint prior distribution for *M* is denoted *P*(*M*).

**Table 1:** Parameters M to be calibrated.

Calibration parameter	Range
***X***_**0**_**(0), *X***_**1**_**(0), *X***_**2**_**(0), *X***_**3**_**(0), *X***_**4**_**(0)**	[0,10^5^]
***D***_***E***_**, *T***_***E***_**, *N***_**E**_**, *S***_**4**_	[0,10^5^]
**log**_**2**_ ***ν***_***D***_**, log**_**2**_ ***ν***_***T***_**, log**_**2**_ ***ν***_***N***_	[–1,1]
***u***_**0**_**,*u***_**14**_	[0,2×10^4^]
${d_{early}}_{0},\;{d_{early}}_{14}$	[0,0.3]
${d_{late}}_{0},\;{d_{late}}_{14}$	[0,0.3]
${d_{HCC}}_{0},\;{d_{HCC}}_{14}$	[0,0.3]

#### Data for model calibration

The posterior distributions of the model’s unknown parameters are proportional to the product of the likelihood function of parameters *M* and the prior distribution *P*(*M*). We used three sets of data in the evaluation of the likelihood of *M*. The first is a set of time-series data, composed of the number of new primary HCC cases recorded annually in Canada, available from Statistics Canada [8]. We denote by *z*_*HCC*_(*t*) the total number of new HCC cases diagnosed in Canada within a given birth cohort in year *t*. The second is also a set of time-series data, composed of the number of hepatitis C diagnoses recorded annually in Canada, available from the Public Health Agency of Canada [9]. In this set, no distinction is made between acute and chronic cases of hepatitis C. We denote by *z*_*hepC*_(*t*) the recorded number of individuals diagnosed with hepatitis C in Canada in the year *t* from within a given birth cohort. These recorded time-series observations are summarized in S4 Table for the years 1999–2013 (years *t* = 0,⋯14) for the three birth cohorts of interest. For notational purposes, we organize the time series observations in year *t* for a given cohort into the vector *z*_*ts*_(*t*) = [*z*_*HCC*_(*t*) *z*_*hepC*_(*t*)]′.

The third set of data used in evaluation of the likelihood of *M* is composed of estimates of the proportions $z_{F_{i}}$ (*i* = 0,⋯,4) of each birth cohort’s total CHC diagnoses falling in fibrosis stages F0 –F4. These estimates, given in S5 Table, are obtained using clinical data from [6]. We organize this data into the vector $z_{F}=[{z_{F}}_{0},\cdots ,{z_{F}}_{4}]\prime$.

#### Model posterior distributions

Letting ***z*** = [*z*_*ts*_(0)′,⋯,*z*_*ts*_(14)′,*z*_*F*_′]′, we denote by *L*(*M*|***z***,*V*) the likelihood function of parameters *M*, which is the joint probability of the data ***z*** given *M* and a fixed value of *V* obtained from the joint distribution *P*(*V*). Derivations of *L*(*M*|***z***,*V*) are given in S2 Appendix. Using *L*(*M*|***z***,*V*), and the joint prior distribution *P*(*M*), we obtain the joint posterior distribution conditional on *V* as

P(M|z,V)∝L(M|z,V)P(M) (15)

For each birth cohort, we estimated the joint posterior distribution *P*(*M*,*V*|***z***) by fitting the model (12) to the cohort’s data vector ***z*** from S4 Table and S5 Table. Samples from this joint distribution were then used to simulate the model (12) and hence evaluate *R*_*x*_(*t*) and ${\bar{D}}_{x}(t)$ using (4) and (5). The joint distribution was generated using Algorithm 1 in S3 Appendix, which is composed of two stages.

**Stage 1 of Algorithm 1**: The first stage consists of two **for** loops, one embedded inside the other. In the *k*^th^ run of the total *K*_1_ = 10^4^ runs of the outer loop, the algorithm first obtains a sample *V*_*k*_ from the distribution *P*(*V*) of the literature-derived parameters *V* (14). Then, the Metropolis-Hastings MCMC algorithm (S3 Appendix) is implemented in the inner loop for *K*_2_ = 10^6^ iterations to ensure that the Markov chain converges to the stationary distribution of the posterior of *M*,*P*(*M*|***z***,*V*_*k*_). At the end of Stage 1, the algorithm outputs *P*(*M*|***z***,*V*_*k*_), *k* = 1,⋯,*K*_1_, the posterior distributions of *M* given ***z*** and *V*_*k*_ for *K*_1_ samples *V*_*k*_ from *P*(*V*).

**Stage 2 of Algorithm 1:** The second stage is composed of a single **for** loop in which we obtain samples from the joint posterior distribution *P*(*M*,*V*|***z***). In each of the *K*_3_ runs of this loop, we first uniformly sample a vector $V_{k}^{*}$ from among the vectors *V*_*k*_ generated in the outer loop of Stage 1, thus yielding a sample from *P*(*V*). Next, we obtain a sample M* from among the samples of the distribution $P(M|z,V_{k}^{*})$ obtained in Stage 1. The combined vector ${\left[V_{k}^{*\prime },M^{*\prime }\right]}^{\prime }$ constitutes a sample from the distribution *P*(*M*,*V*|***z***).

Algorithm 1 was used to obtain samples from the joint posterior distribution *P*(*M*,*V*|***z***) for each of the three cohorts listed in S4 Table. Distributions for the CHC prevalence rate *R*_*x*_(*t*) and the percentage of CHC population undiagnosed ${\bar{D}}_{x}(t)$ were then obtained for each cohort by repeatedly sampling the cohort’s joint posterior distributions, using the samples to simulate the model, and then evaluating (4) and (5).

## Results

We next summarize the results obtained for each cohort.

### 1) CHC in Canada for pre-1945 births cohort

**Fig 3:**
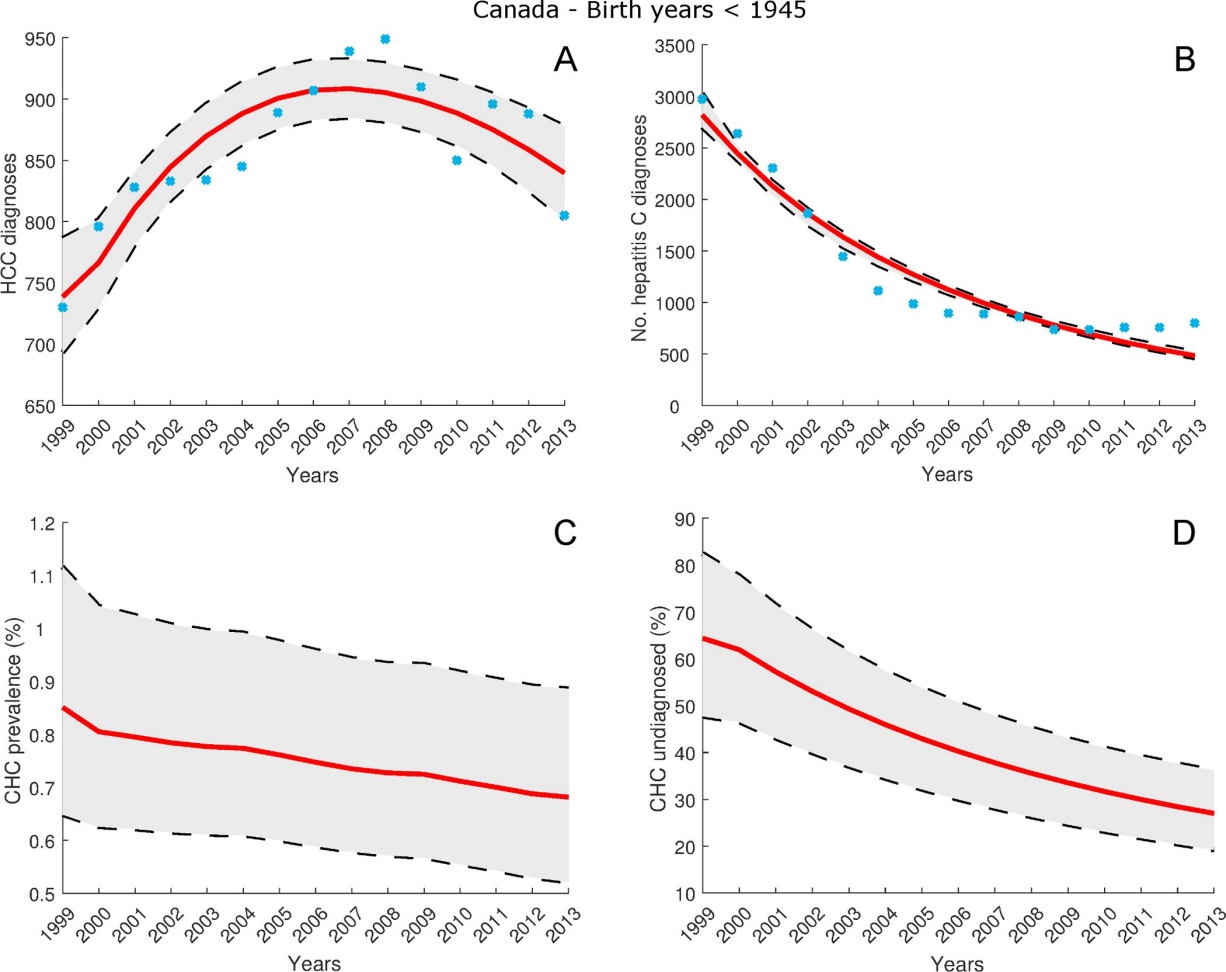
Estimates for cohort of individuals born before 1945, for years 1999–2013, from model (12), (13) calibrated using data listed in S4 Table and S5 Table. Blue circles denote observed annual numbers of HCC and hepatitis C diagnoses for this cohort. A: Model estimates of total number of HCC diagnoses. B: Model estimates of total number of hepatitis C diagnoses. C: Model estimates of CHC prevalence rate *R*_*x*_(*t*). D: Model estimates of proportion of CHC population undiagnosed ${\bar{D}}_{x}(t)$.

For the cohort of individuals across Canada who were born before 1945, Fig 3A and Fig 3B show the estimates by model (12), (13) of the HCC and hepatitis C diagnosis data listed in S4 Table. The mean estimates fit the HCC diagnosis data with *R*^2^ = 0.76, and the hepatitis C diagnosis data with *R*^2^ = 0.93. Fig 3C gives the estimate of hepatitis C prevalence rate *R*_*x*_ in 2013 as 0.68% (95% CI: 0.52% - 0.89%). From Fig 3D, the percentage of CHC cases undiagnosed ${\bar{D}}_{x}$ in 2013 was 27.0% (95% CI: 19.3% - 36.1%). The estimated mean number of total CHC cases and undiagnosed CHC cases is given in S4 Appendix.

#### CHC in 1945–1964 births cohort

**Fig 4:**
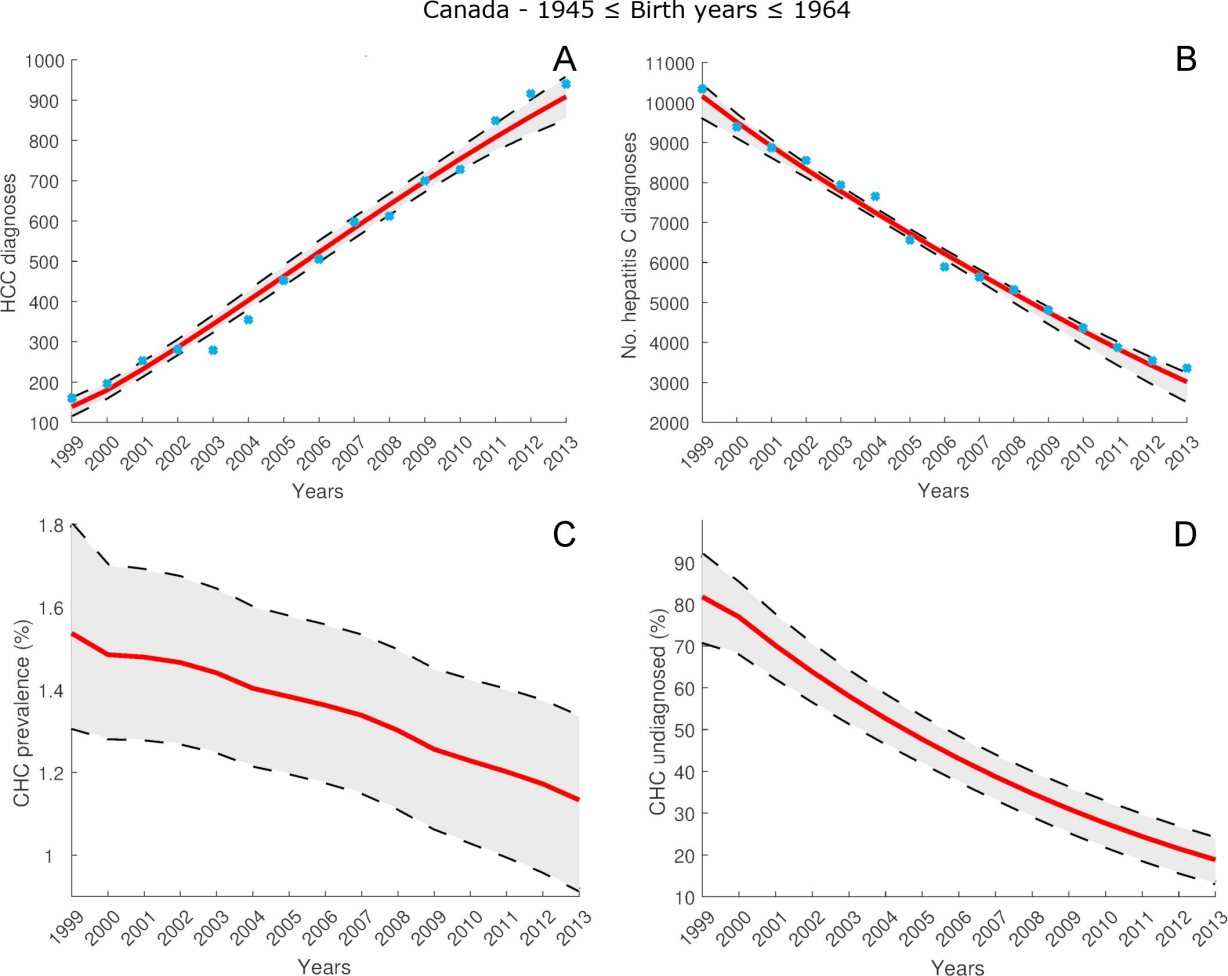
Estimates for cohort of individuals born between 1945 and 1964, for years 1999–2013, from model (12), (13) calibrated using data listed in S4 Table and S5 Table. Blue circles denote observed annual numbers of HCC and hepatitis C diagnoses for this cohort. A: Model estimates of total number of HCC diagnoses. B: Model estimates of total number of hepatitis C diagnoses. C: Model estimates of CHC prevalence rate *R*_*x*_(*t*). D: Model estimates of proportion of CHC population undiagnosed ${\bar{D}}_{x}(t)$.

For the cohort of individuals across Canada who were born between 1945 and 1964, Fig 4A and Fig 4B show the estimates by model (12), (13) of the HCC and hepatitis C diagnosis data listed in S4 Table. The mean estimates fit the HCC diagnosis data with *R*^2^ = 0.98, and the hepatitis C diagnosis data with *R*^2^ = 0.99. Fig 4C gives the estimate of hepatitis C prevalence rate *R*_*x*_ in 2013 as 1.13% (95% CI: 0.92% - 1.33%). From Fig 4D, the percentage of CHC cases undiagnosed ${\bar{D}}_{x}$ in 2013 was 18.8% (95% CI: 13.3% - 23.9%). The estimated mean number of total CHC cases and undiagnosed CHC cases is given in S4 Appendix.

### CHC in Canada for post-1964 birth years cohort

**Fig 5:**
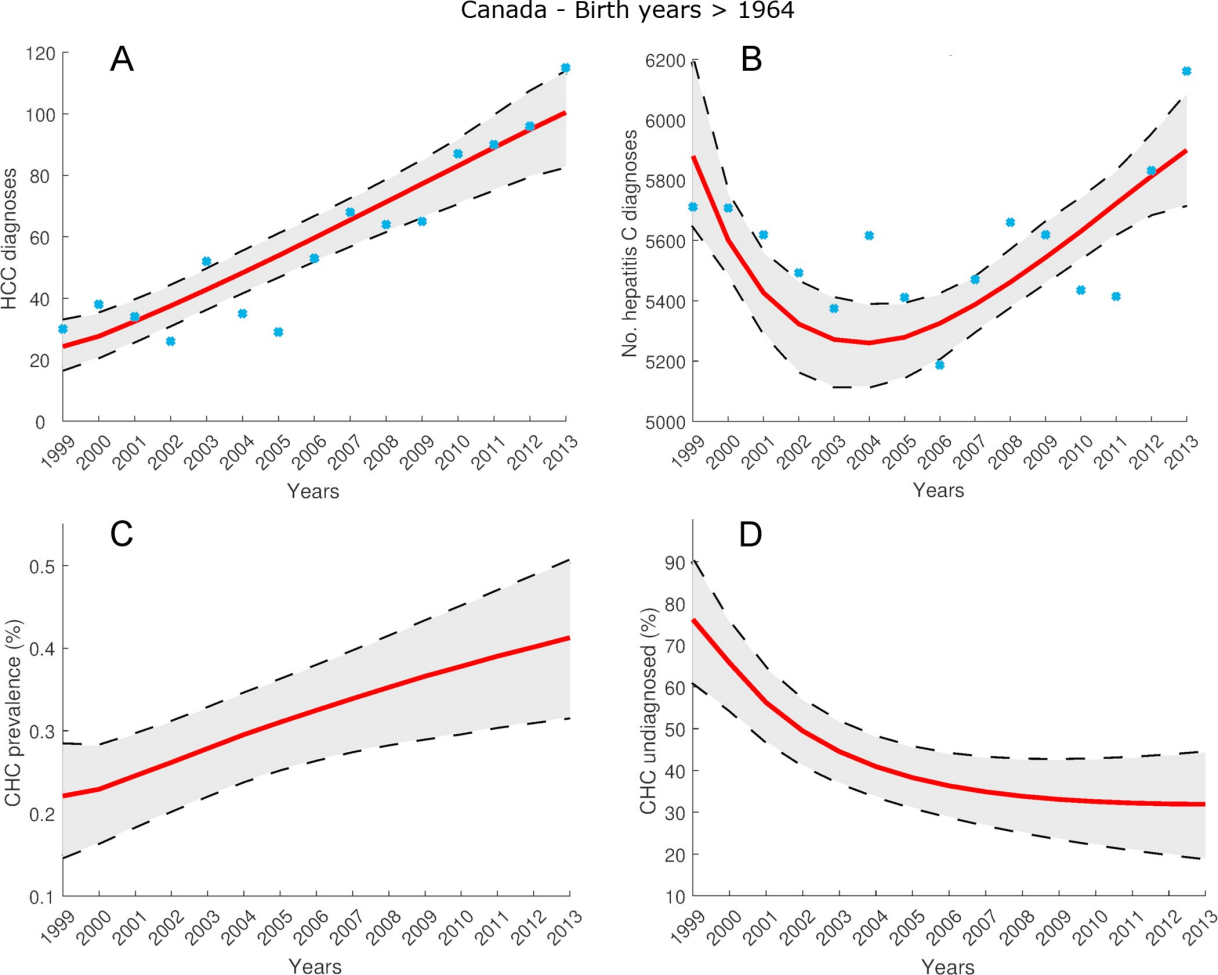
Estimates for cohort of individuals born after 1964, for years 1999–2013, from model (12), (13) calibrated using data listed in S4 Table and S5 Table. Blue circles denote observed annual numbers of HCC and hepatitis C diagnoses for this cohort. A: Model estimates of total number of HCC diagnoses. B: Model estimates of total number of hepatitis C diagnoses. C: Model estimates of CHC prevalence rate *R*_*x*_(*t*). D: Model estimates of proportion of CHC population undiagnosed ${\bar{D}}_{x}(t)$.

For the cohort of individuals across Canada who were born after 1964, Fig 5A and Fig 5B show the estimates by model (12), (13) of the HCC and hepatitis C diagnosis data listed in S4 Table. The mean estimates fit the HCC diagnosis data with *R*^2^ = 0.85, and the hepatitis C diagnosis data with *R*^2^ = 0.28. Fig 5C gives the estimate of hepatitis C prevalence rate *R*_*x*_ in 2013 as 0.41% (95% CI: 0.32% - 0.51%). From Fig 5D, the percentage of CHC cases undiagnosed ${\bar{D}}_{x}$ in 2013 was 31.9% (95% CI: 19.1% - 44.2%). The estimated mean number of total CHC cases and undiagnosed CHC cases is given in S4 Appendix.

#### CHC in Canada averaged over all birth cohorts

**Fig 6:**
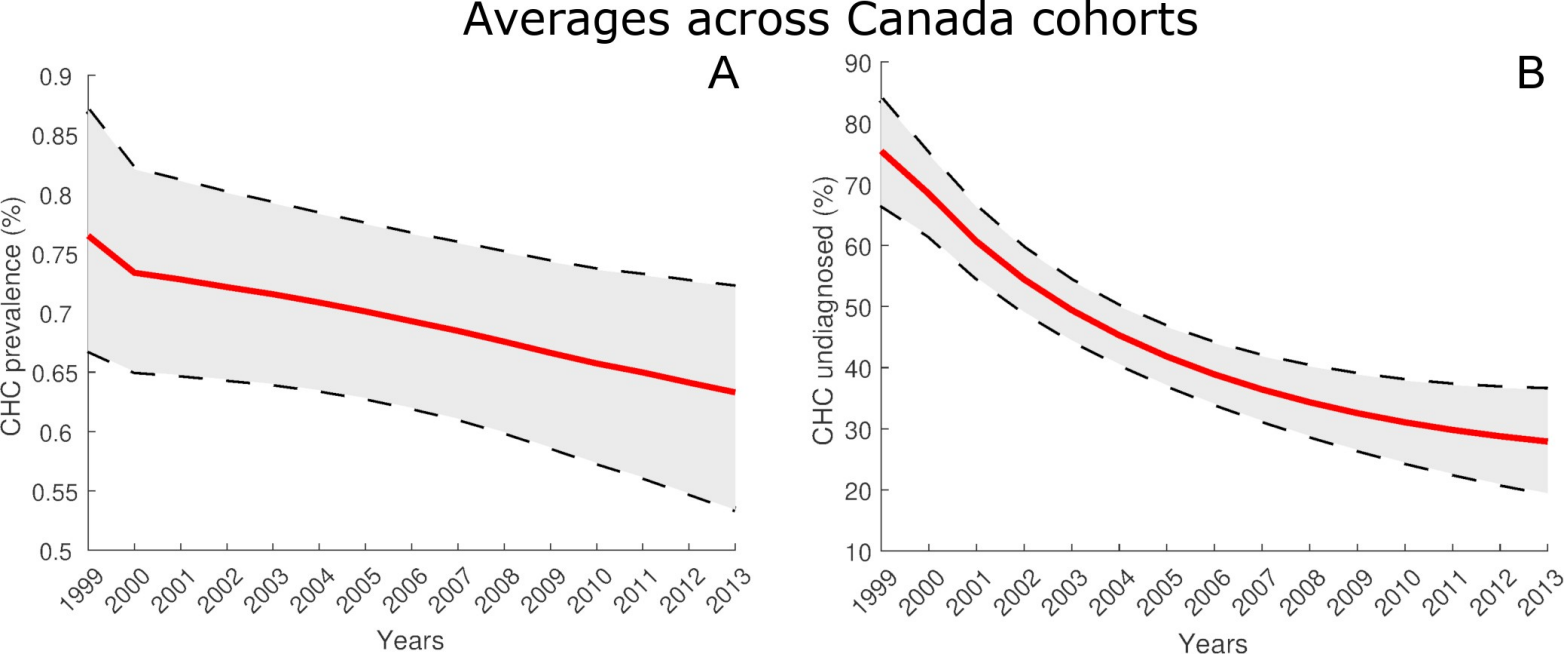
Cohort-averaged estimates of prevalence rate and proportion of CHC population undiagnosed. A: Estimates for years 1999–2013 of CHC prevalence rate in Canada rate averaged over all birth cohorts. B: Estimates for years 1999–2013 of proportion of CHC population undiagnosed averaged over all birth cohorts.

Fig 6A shows the prevalence rate estimate for Canada averaged over all birth cohorts. The CHC prevalence rate estimate in 2013 was 0.63% (95% CI: 0.53% - 0.72%). Fig 6B shows the percentage of CHC cases in Canada that were undiagnosed, averaged over all birth cohorts. The percentage of CHC cases undiagnosed in 2013 was estimated to be 27.1% (95% CI: 19.3% - 36.1%).

### Sensitivity analysis

**Fig 7:**
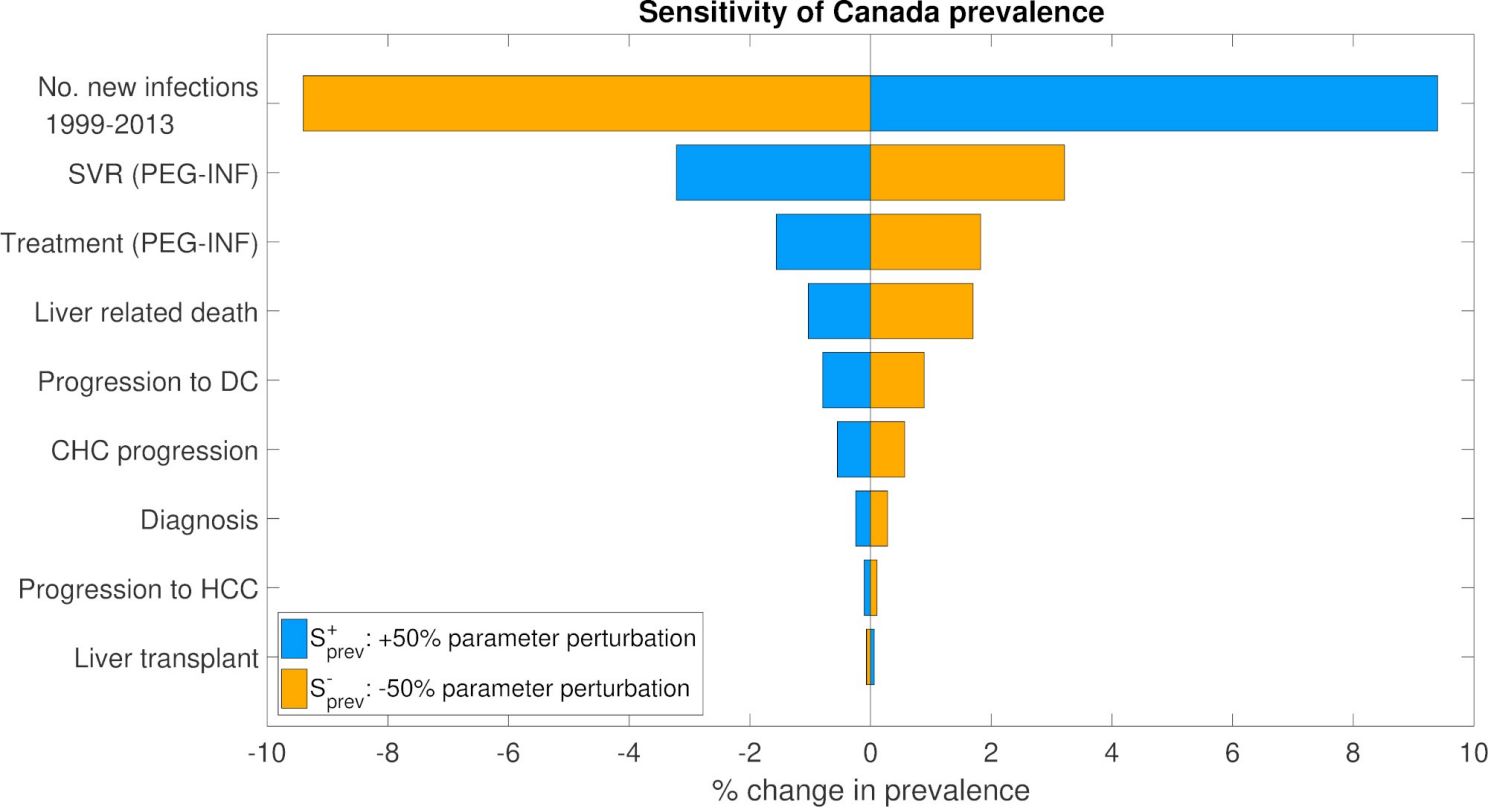
One-way sensitivity analysis for estimated prevalence rate, averaged over all birth cohorts.

**Fig 8:**
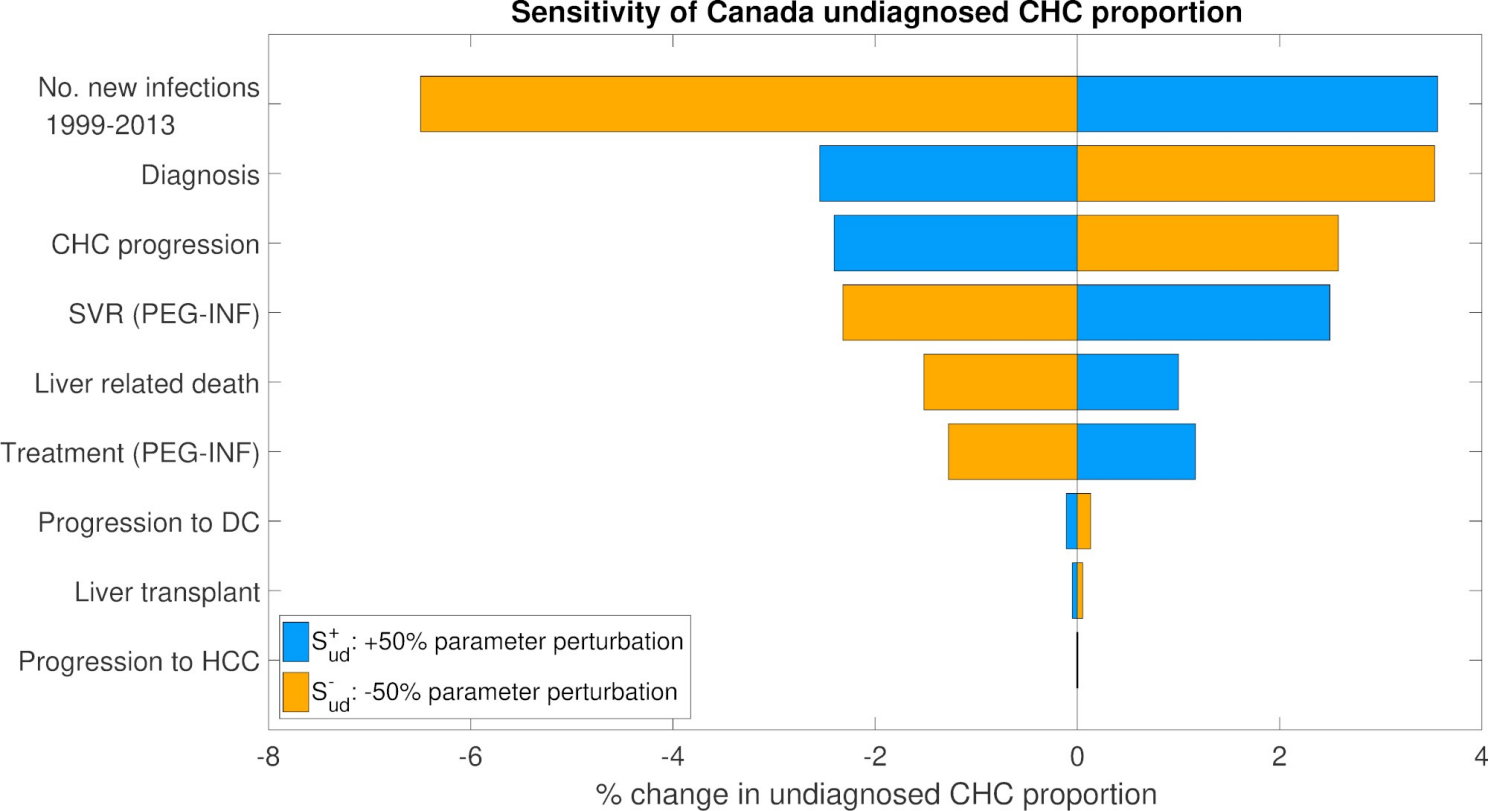
One-way sensitivity analysis for estimated proportion of CHC-infected population undiagnosed, averaged all birth cohorts.

A one-way sensitivity analysis of the calibrated models was conducted to determine the parameter categories causing the most variation in estimates of prevalence rate and undiagnosed CHC proportion. The parameters tested were grouped into nine categories, listed in S6 Table. The parameters in each group were varied together by +/-50%, and the effects of these perturbations on the areas under the curves (AUC) of *R*_*x*_ and ${\bar{D}}_{x}$ between years 1999 and 2013 were evaluated. The groups were then ranked according to how sensitive the cohort-averaged estimates of *R*_*x*_ and ${\bar{D}}_{x}$ AUCs were to each. The CHC prevalence rate (*R*_*x*_) AUC, averaged over all three birth cohorts, was most sensitive to perturbations in the number of new infections (Fig 7). A perturbation of +50% in the yearly number of new infections yielded a change of +9.4% in the cohort-averaged *R*_*x*_ AUC. The AUC for the proportion of the CHC population undiagnosed (${\bar{D}}_{x}$), averaged over the three birth cohorts, was also most sensitive to perturbations to the number of new infections, followed by perturbations to the annual probability of CHC diagnosis (Fig 8). Here, a perturbation of -50% in the number of new infections yielded a change of -6.50% in the cohort-averaged ${\bar{D}}_{x}$ AUC, whereas a perturbation of -50% in the annual probability of CHC diagnosis yielded a corresponding increase of +3.53%.

## Discussion

CHC often progresses silently and asymptomatically until late in its course. Recently, hepatologists have issued hepatitis C screening recommendations for Canadian baby-boomers [20]. However, hepatitis C screening in Canada has only been recommended for high-risk individuals by the Public Health Agency of Canada [21], partly due to the uncertain prospective financial burden of a publicly funded screening and treatment policy. An accurate estimate of the national prevalence rate of CHC and the proportion undiagnosed is therefore needed to reliably predict the disease burden and to perform economic evaluations of population screening and disease eradication strategies.

The aim of this work was to establish a mathematical framework to estimate CHC populations of interest. Starting with a well-established hepatitis C natural history model, our study proposed a method with which to calibrate the model using available diagnosis data. As a case study, we demonstrated the use of the framework in estimating Canada’s CHC prevalence and the proportion of the Canadian CHC population that is undiagnosed.

The averaged prevalence rate of CHC in Canada was estimated to be 0.63% (95% CI: 0.53% - 0.72%) in 2013. The averaged percentage of CHC cases undiagnosed in Canada in 2013 was estimated to be 27.1% (95% CI: 19.3% - 36.1%). Among the Canadian baby boomer generation (birth cohort 1945–1964), the CHC prevalence rate was estimated to be falling between 1999 and 2013, and at 1.13% (95% CI: 0.92% - 1.33%) with 18.8% (95% CI: 13.3% - 23.9%) of individuals with CHC undiagnosed in 2013. On the other hand, among the younger birth cohort (births years > 1964), the CHC prevalence rate was estimated to be rising between 1999 and 2013, and at 0.41% (95% CI: 0.32% - 0.51%) in 2013 with 31.9% (95% CI: 19.1% - 44.2%) of individuals with CHC undiagnosed in 2013. The falling trend over 1999–2013 in the baby boomer cohort is to be expected since a greater number of CHC infected individuals in this cohort have already progressed to advanced liver disease stage, in which the mortality rate is high. The prevalence rate in the younger cohort is low but shows a rising trend over the 1999–2013 period. With the baby-boomer infected population falling and the lower-prevalence rate younger cohort forming an increasingly large proportion of the Canadian population over 1999–2013, the overall trend in the CHC prevalence rate was a downward one, as seen in Fig 6A. As more data becomes available with time, it can easily be incorporated into the modeling framework of this paper to yield more up-to-date estimates of CHC prevalence.

This study’s estimates were based on 1) a comprehensive mathematical framework that is built on a validated hepatitis C natural history model; and 2) an approach that incorporates the sources of uncertainty inherent to hepatitis C natural history. Compared with previous results, our national CHC prevalence rate estimate for 2007 is 0.68% (95% CI 0.61% - 0.76%) and is therefore lower than the 2007 estimate by Remis of 0.78% [3]. On the other hand, our 2011 prevalence rate estimate is 0.65% (95% CI 0.56–0.73) and is therefore in agreement with the 0.64–0.71% estimate for 2011 reported in the Canada Communicable Disease Report [5]. When compared with the seroprevalence rate of 0.5% (range 0.3–0.9%) estimated from the Canadian Health Measures Survey from 2007 to 2011 [22], our model estimated a higher prevalence rate. Estimates from the Canadian Health Measures Survey may be biased towards the low side as only regular households were surveyed while marginalized and high-needs populations may have been undersampled [22].

The one-way sensitivity analyses identified those parameters which had the greatest impact on the model estimates. Both the prevalence rate estimates and the undiagnosed CHC proportion were found to be most sensitive to the annual number of new infections. The undiagnosed CHC proportion was additionally found to be sensitive to the annual probability of CHC diagnosis. These particular parameters are the least well known of all the parameters of the model. As shown in S5 Appendix, there are significant differences between the posterior and prior distributions of these parameters, indicating their sensitivity to the observed numbers of diagnosed HCC and hepatitis C cases. This highlights the importance of high quality calibration data for the accurate calibration of these parameters and the subsequent generation of reliable model estimates. The sensitivity analyses therefore point to where additional research effort should be directed to improve estimate accuracy. This is particularly useful information for health economists who may wish to use this model for a ‘value of information’ analysis [23,24].

With respect to the backcalculation estimate in [5], our model quantifies the hepatitis C epidemic in more detail, in several respects: 1) it models the adoption of treatment among CHC patients as well as the probability of achievement of a sustained virologic response (SVR), a state in which HCV patients are considered effectively cured of the viral infection, 2) it accounts for the dependence, on viral genotype, of the rates of treatment, SVR and CHC progression, 3) it models the progression of CHC to advanced liver disease, and, 4) it models the heightened rate of mortality among younger CHC population cohorts [3,15,16]. Furthermore, a wider and more up-to-date set of data is used in this study with respect to [5]: first, our framework quantifies the proportion of HCC diagnosis numbers (used in the calibration) that are attributable to CHC. Second, Trubnikov et al. [5] made no use of statistics on liver fibrosis distributions at diagnosis as was done in this work. Finally, the Bayesian approach we adopt in calibrating the model returns prevalence estimates in the form of posterior probability distributions that reflect the parametric uncertainty in the model.

Although the model used in this study is more comprehensive than those used in [3] and [5], the framework we have presented is also subject to some limitations. The model relied on the total number of HCC cases diagnosed each year as one of the observables. Because progression from a new HCV infection to HCC may occur over a period of 20–40 years [25], the model may underestimate the prevalence of CHC in the youngest cohort. A further limitation arises from uncertainty in the calibration data. These data were derived from public databases which are subject to the usual limitations of under-reporting, incompleteness, duplication and sampling bias. In future work, uncertainty in our estimates can be reduced with calibration data that i) report the annual numbers of HCC cases that are confirmed as being CHC-induced and ii) decompose the annual numbers of hepatitis C diagnoses into acute, resolved and chronic cases.

Using a Bayesian Markov Chain Monte Carlo approach, this paper has presented a method for the estimation of CHC populations of interest from a hepatitis C natural history model calibrated using publicly available data on the occurrence of HCV-related events. One of the advantages of this framework is its ability to incorporate multiple sources of uncertainty into the model in arriving at its estimates. This factor makes the estimates more robust and is essential for decision-making [26]. In future work, this approach can be generalized and used to produce region-specific estimates.

## Supporting information

S1 Table Literature-derived model parameters. Annual probabilities of progression to CHC, progression between fibrosis stages, progression to DC, and advanced liver disease progression.
(PDF)

S2 Table Annual probabilities of treatment adoption by fibrosis stage and viral genotype for the population of Canada. (PDF)

S3 Table Percentage of HCC cases induced by CHC. (PDF)

S4 Table Diagnosis data for model calibration. HCC diagnoses, hepatitis C diagnoses, and population, by cohort, for Canada, 1999–2013. Year *t* = 0 corresponds to year 1999, year *t* = 14 corresponds to year 2013.
(PDF)

S5 Table Distribution of fibrosis levels for each birth cohort. (PDF)

S6 Table Parameter groups perturbed in one-way sensitivity analysis. (PDF)

S1 Appendix Details of state transition model equations. (PDF)

S2 Appendix Construction of the model likelihood function. (PDF)

S3 Appendix Details of model fitting algorithm. (PDF)

S4 Appendix Mean estimates of total numbers of CHC cases and undiagnosed CHC cases. (PDF)

S5 Appendix Prior and posterior distributions of year 2013 number of new infections and probabilities of diagnosis. (PDF)
