# Supplementary material for: A model-based framework for chronic hepatitis C prevalence estimation
Source: PLoS One. 2019 Nov 21;14(11):e0225366. doi: 10.1371/journal.pone.0225366 (PMC6874092; doi:10.1371/journal.pone.0225366)
Supplement: S1 Table — Annual probabilities of progression to CHC, progression between fibrosis stages, progression to DC, and advanced liver disease progression. (PDF) [file pone.0225366.s001.pdf]

Parameter	Transition, reference	Annual probability mean (range)
q_{chr}	Acute →F0, [10]	0.70 (0.55-0.85)
q_{01}	F0→F1, [4]	0.117 (0.104 - 0.130)
q_{12}	F1→F2, [4]	0.085 (0.075 - 0.096)
q_{23}	F2→F3, [4]	0.120 (0.109 - 0.133)
q_{34}	F3→F4, [4]	0.116 (0.104 - 0.129)
d_{DC}	F4 → DC, [12]	0.035 (0.027 - 0.043)
d_{DC-SVR}	F4 (SVR) → DC, [12]	0.002 (0.0001 - 0.005)
a_1	HCC→LT, [23]	0.033 (0.017 - 0.049)
a_2	HCC →LD, [24]	0.411 (0.31 - 0.51)
a_3	DC→LT, [23]	0.033 (0.017 - 0.049)
a_4	DC →LD, [25]	0.216 (0.162 - 0.27)
a_5	LT → LD within one year, [26]	0.142 (0.124 - 0.159)
a_6	LT → LD after one year, [26]	0.034 (0.024 - 0.043)

S1 Table: Literature-derived model parameters. Annual probabilities of progression to CHC, progression between fibrosis stages, progression to DC, and advanced liver disease progression.
