# Supplementary material for: A model-based framework for chronic hepatitis C prevalence estimation
Source: PLoS One. 2019 Nov 21;14(11):e0225366. doi: 10.1371/journal.pone.0225366 (PMC6874092; doi:10.1371/journal.pone.0225366)
Supplement: S2 Table — (PDF) [file pone.0225366.s002.pdf]

	Annual probability of treatment t_i		
**Fibrosis stage**	**Genotype 1**	**Genotypes 2,3**	**Genotypes 4,5,6**
F0	0.03 (0.02 - 0.04)	0.04 (0.03 - 0.06)	0.05 (0.03 - 0.06)
F1	0.05 (0.03 - 0.06)	0.07 (0.05 - 0.11)	0.05 (0.03 - 0.06)
F2	0.05 (0.03 - 0.06)	0.07 (0.05 - 0.11)	0.05 (0.03 - 0.06)
F3	0.05 (0.03 - 0.08)	0.07 (0.05 - 0.12)	0.05 (0.03 - 0.06)
F4	0.06 (0.04 - 0.10)	0.06 (0.04 - 0.10)	0.06 (0.04 - 0.09)

S2 Table: Annual probabilities of treatment adoption by fibrosis stage and viral genotype for the population of Canada.
