# Supplementary material for: A model-based framework for chronic hepatitis C prevalence estimation
Source: PLoS One. 2019 Nov 21;14(11):e0225366. doi: 10.1371/journal.pone.0225366 (PMC6874092; doi:10.1371/journal.pone.0225366)
Supplement: S3 Table — (PDF) [file pone.0225366.s003.pdf]

Birth cohort	Percentage of HCC cases induced by CHC ($\mathbf{100 \times c_{HCC}}$)
Births before 1945	13 (7-20)
Births between 1945 and 1964	46 (23-69)
Births after 1964	6 (4-8)

S3 Table: Percentage of HCC cases induced by CHC.
