# Supplementary material for: A model-based framework for chronic hepatitis C prevalence estimation
Source: PLoS One. 2019 Nov 21;14(11):e0225366. doi: 10.1371/journal.pone.0225366 (PMC6874092; doi:10.1371/journal.pone.0225366)
Supplement: S4 Table — HCC diagnoses, hepatitis C diagnoses, and population, by cohort, for Canada, 1999–2013. Year t = 0 corresponds to year 1999, year t = 14 corresponds to year 2013. (PDF) [file pone.0225366.s004.pdf]

	Birth years < 1945			1945 ≤ Birth years ≤ 1964			Birth years ≥ 1965		
**Year** t	**HCC diagnoses** $z_{HCC}(t)$	**Hep C diagnoses** $z_{hepC}(t)$	**Population (×1000)** $p_n(t)$	**HCC diagnoses** $z_{HCC}(t)$	**Hep C diagnoses** $z_{hepC}(t)$	**Population (×1000)** $p_n(t)$	**HCC diagnoses** $z_{HCC}(t)$	**Hep C diagnoses** $z_{hepC}(t)$	**Population (×1000)** $p_n(t)$
0	730	2974	6385	160	10340	9460	30	5711	14434
1	796	2639	6217	196	9390	9426	38	5708	14917
2	828	2305	6049	253	8870	9390	34	5619	15452
3	833	1865	5882	281	8551	9373	26	5493	15967
4	834	1448	5678	279	7938	9408	52	5374	16410
5	845	1115	5444	355	7655	9506	35	5616	16838
6	889	987	5276	452	6562	9467	29	5411	17344
7	907	897	5109	505	5889	9415	53	5187	17884
8	939	890	4929	598	5626	9369	68	5470	18420
9	949	861	4717	612	5321	9394	64	5660	18957
10	910	739	4475	700	4807	9479	65	5619	19489
11	850	737	4303	728	4364	9421	87	5435	20081
12	896	759	4121	849	3873	9340	90	5415	20666
13	888	758	3944	916	3545	9274	96	5832	21298
14	805	801	3737	940	3356	9278	115	6161	21891

S4 Table: Diagnosis data for model calibration. HCC diagnoses, hepatitis C diagnoses, and population, by cohort, for Canada, 1999-2013. Year $t = 0$ corresponds to year 1999, year $t = 14$ corresponds to year 2013.
