# Supplementary material for: A model-based framework for chronic hepatitis C prevalence estimation
Source: PLoS One. 2019 Nov 21;14(11):e0225366. doi: 10.1371/journal.pone.0225366 (PMC6874092; doi:10.1371/journal.pone.0225366)
Supplement: S5 Table — (PDF) [file pone.0225366.s005.pdf]

	Fibrosis distribution z_{F_i} (%)		
**Fibrosis stage i**	**Births years < 1945**	**1945 ≤ Birth years ≤ 1964**	**Birth years ≥ 1965**
0	0.5	4.0	13.5
1	20.0	30.5	35.8
2	20.0	30.5	35.8
3	30.5	20.5	9.5
4	29.0	14.5	5.5

S5 Table: Distribution of fibrosis levels for each birth cohort.
