# Supplementary material for: A model-based framework for chronic hepatitis C prevalence estimation
Source: PLoS One. 2019 Nov 21;14(11):e0225366. doi: 10.1371/journal.pone.0225366 (PMC6874092; doi:10.1371/journal.pone.0225366)
Supplement: S6 Table — (PDF) [file pone.0225366.s006.pdf]

**Parameter groups**	**Model parameters**
CHC progression annual probability	$q_{01}, q_{12}, q_{23}, q_{34}$
Treatment annual probability	t_0, t_1, t_2, t_3, t_4
SVR annual probability	s_0, s_1, s_2, s_3, s_4
Diagnosis annual probability	$d_{early_0}, d_{early_{14}}, d_{late_0}, d_{late_{14}}$
Progression to HCC annual probability	$d_{HCC_0}, d_{HCC_{14}}$
Progression to DC annual probability	$d_{DC}, d_{DC_{SVR}}$
Liver transplant annual probability	a_1, a_3
Liver related death annual probability	a_2, a_4, a_5, a_6
Number of new infections	u_0, u_{14}

S6 Table: Parameter groups perturbed in one-way sensitivity analysis.
