# Supplementary material for: A model-based framework for chronic hepatitis C prevalence estimation
Source: PLoS One. 2019 Nov 21;14(11):e0225366. doi: 10.1371/journal.pone.0225366 (PMC6874092; doi:10.1371/journal.pone.0225366)
Supplement: S1 Appendix — (PDF) [file pone.0225366.s007.pdf]

S1 Appendix - Details of state transition model equations

We use the following example to illustrate how the state transition probabilities of the model are derived.

Example: With $d_r(t)$ being the annual background probability of death, we let $\sigma(t) = 1 - d_r(t)$, which is the annual probability that an individual will not die of CHC-related causes. The number of individuals who, at the beginning of the year 2000, were in the undiagnosed F0 stage of CHC (stage X_0) can be estimated to be equal to a proportion $q_{chr}(1 - d_a)\sigma(t)$ of $u(1999)$ (the number of new infections in 1999) plus a proportion $\alpha_0^X(t) = \left(1 - q_{0,1}\right)(1 - d_0)\sigma(t)$ of $X_0(1999)$ (the number of individuals in state X_0 in 1999). Here, q_{chr} is the probability that a new hepatitis C infection will become chronic, d_a is the probability that an individual with an acute infection will be diagnosed before the infection turns chronic, q_{01} is the annual probability that an undiagnosed individual in fibrosis stage F0 will progress to fibrosis stage F1, and d_0 is the annual probability that an undiagnosed individual in fibrosis stage F0 will be diagnosed with CHC. Therefore:

$$X_0(2000) = q_{chr}(1 - d_a)\sigma(t)\, u(1999) + \alpha_0^X(t)X_0(1999)$$

The first term represents the number of individuals who became infected with HCV in 1999 who survived until year 2000, and who remained undiagnosed throughout 1999, and whose infection became chronic between the 1999 and 2000. The second term represents the number of individuals who had CHC at stage F0 in 1999, and who survived until 2000, and who remained undiagnosed throughout 1999, and whose infection did not progress from F0 to F1 in 1999.

From the state transition model illustrated in Fig 1, we similarly arrive at the following expressions, which are functions of parameters in S1 Table:

Parameter formula	Description
$\alpha_i^X(t) = \left(1 - q_{i,i+1}\right)(1 - d_i)\sigma(t)$	Probability that an individual in state X_i (i=0,1,2,3) in year t will remain in state X_i in year $t + 1$.
$\beta_i^X(t) = q_{i,i+1}(1 - d_i)\, \sigma(t)$	Probability that an individual in state X_i (i=0,1,2,3) in year t will progress in liver fibrosis, transitioning to state X_{i+1} in year $t + 1$.

$\gamma^X(t) = (1 - d_{DC} - d_{HCC} - d_4)\sigma(t)$	Probability that an individual in state X_4 in year t will remain in state X_4 in year $t+1$.
$\delta_i^D(t) = (1 - q_{i,i+1})d_i\sigma(t)$	Probability that an individual in state X_i (i=0,1,2,3) in year t will be diagnosed, transitioning to state D_i in year $t+1$.
$\tau_i^D(t) = q_{i,i+1}d_i\sigma(t)$	Probability that an individual in state X_i (i=0,1,2,3) in year t will progress in liver fibrosis and be diagnosed, transitioning to state D_{i+1} in year $t+1$.
$\varepsilon^D(t) = d_4\sigma(t)$	Probability that an individual in state X_4 in year t will be diagnosed, transitioning to state D_4 in year $t+1$.
$\alpha_i^D(t) = (1 - q_{i,i+1})(1 - t_i(v))\,\sigma(t)$	Probability that an individual in state D_i (i=0,1,2,3) in year t will remain in state D_i in year $t+1$ (for viral genotype v).
$\beta_i^D(t) = q_{i,i+1}(1 - t_i(v))\,\sigma(t)$	Probability that an individual in state D_i (i=0,1,2,3) in year t will progress in liver fibrosis, transitioning to state D_{i+1} in year $t+1$ (for viral genotype v).
$\gamma^D(t) = (1 - d_{DC} - d_{HCC} - t_4(v))\sigma(t)$	Probability that an individual in state D_4 in year t will remain in state D_4 in year $t+1$ (for viral genotype v).
$\delta_i^T(t) = (1 - q_{i,i+1})t_i(v)\,\sigma(t)$	Probability that an individual in state D_i (i=0,1,2,3) in year t will be treated, transitioning to state T_i in year $t+1$ (for viral genotype v).
$\tau_i^T(t) = q_{i,i+1}t_i(v)\sigma(t)$	Probability that an individual in state D_i (i=0,1,2,3) in year t will progress in liver fibrosis and be treated, transitioning to state T_{i+1} in year $t+1$ (for viral genotype v).
$\varepsilon^T(t) = t_4(v)\,\sigma(t)$	Probability that an individual in state D_4 in year t will be diagnosed, transitioning to state T_4 in year $t+1$ (for viral genotype v).
$\alpha_i^T(t) = 0$	Probability that an individual in state T_i (i=0,1,2,3) in year t will remain in state T_i in year $t+1$.
$\beta_i^T(t) = 0$	Probability that an individual in state T_i (i=0,1,2,3) in year t will progress in liver fibrosis, transitioning to state T_{i+1} in year $t+1$.
$\gamma^T(t) = 0$	Probability that an individual in state T_4 in year t will remain in state T_4 in year $t+1$.
$\delta_i^S(t) = s_i(v)\sigma(t)$	Probability that an individual in state T_i (i=0,1,2,3) in year t will show SVR, transitioning to state S_i in year $t+1$ (for viral genotype v).
$\tau_i^S(t) = 0$	Probability that an individual in state T_i (i=0,1,2,3) in year t will progress in liver fibrosis and show SVR, transitioning to state S_{i+1} in year $t+1$.
$\varepsilon^S(t) = s_4(v)\sigma(t)$	Probability that an individual in state T_4 in year t will show SVR, transitioning to state S_4 in year $t+1$ (for viral genotype v)..
$\alpha_i^S(t) = \sigma(t)$	Probability that an individual in state S_i (i=0,1,2,3) in year t will remain in state S_i in year $t+1$.
$\beta_i^S(t) = 0$	Probability that an individual in state S_i (i=0,1,2,3) in year t will progress in liver fibrosis, transitioning to state S_{i+1} in year $t+1$.
$\gamma^S(t) = (1 - d_{DC-SVR} - d_{HCC-SVR})\sigma(t)$	Probability that an individual in state S_4 in year t will remain in state S_4 in year $t+1$.
$\delta_i^N(t) = (1 - s_i(v))\sigma(t)$	Probability that an individual in state T_i (i=0,1,2,3) in year t will not show SVR, transitioning to state N_i in year $t+1$ (for viral genotype v).
$\tau_i^N(t) = 0$	Probability that an individual in state T_i (i=0,1,2,3) in year t will progress in liver fibrosis and not show SVR, transitioning to state N_{i+1} in year $t+1$.
$\varepsilon^N(t) = (1 - d_{DC} - d_{HCC} - s_4(v))\sigma(t)$	Probability that an individual in state T_4 in year t will not show SVR, transitioning to state N_4 in year $t+1$ (for viral genotype v)..
$\alpha_i^N(t) = (1 - q_{i,i+1})\sigma(t)$	Probability that an individual in state N_i (i=0,1,2,3) in year t will remain in state N_i in year $t+1$.

$\beta_i^N(t) = q_{i,i+1}\sigma(t)$	Probability that an individual in state N_i (i=0,1,2,3) in year t will progress in liver fibrosis, transitioning to state N_{i+1} in year $t+1$.
$\gamma^N(t) = (1 - d_{DC} - d_{HCC})\sigma(t)$	Probability that an individual in state N_4 in year t will remain in state N_4 in year $t+1$.
$b_{DC}(t) = d_{DC}\sigma(t)$	Probability that an individual in state X_4, D_4 or N_4 in year t will develop decompensated cirrhosis, transitioning to state DC in year $t+1$.
$b_{DC-SVR}(t) = d_{DC-SVR}\sigma(t)$	Probability that an individual in state S_4 in year t will develop decompensated cirrhosis, transitioning to state DC in year $t+1$.
$b_{HCC}(t) = d_{HCC}\sigma(t)$	Probability that an individual in state X_4, D_4 or N_4 in year t will develop hepatocellular carcinoma, transitioning to state HCC in year $t+1$.
$b_{HCC-SVR}(t) = d_{HCC-SVR}\sigma(t)$.	Probability that an individual in state S_4 in year t will develop hepatocellular carcinoma, transitioning to state HCC in year $t+1$.
$A_{DC}(t) = (\sigma(t) - a_4)(1 - a_3)$	Probability that an individual in state DC in year t will remain in state DC in year $t+1$.
$A_{HCC}(t) = (\sigma(t) - a_2)(1 - a_1)$	Probability that an individual in state HCC in year t will remain in state HCC in year $t+1$.
$A_{PT}(t) = \sigma(t) - a_6$	Probability that an individual in state PT in year t will remain in state PT in year $t+1$.
$B_{LT-DC}(t) = (\sigma(t) - a_4)a_3$	Probability that an individual in state DC in year t will receive a liver transplant, transitioning to state LT in year $t+1$.
$B_{LT-HCC}(t) = (\sigma(t) - a_2)a_1$	Probability that an individual in state HCC in year t will receive a liver transplant, transitioning to state LT in year $t+1$.
$B_{PT}(t) = \sigma(t) - a_5$	Probability that an individual that receives a liver transplant (state LT) in year t will survive, transitioning to state PT in year $t+1$.

The above state transition probabilities feed directly into the state transition matrices $A_X(t), A_D(t), A_T(t), A_S(t), A_N(t)$ and $B_D(t), B_T(t), B_S(t), B_N(t)$, which take the form

$$A_{[\cdot]}(t) = \begin{bmatrix} \alpha_0^{[\cdot]} & 0 & 0 & 0 & 0 \\ \beta_0^{[\cdot]} & \alpha_1^{[\cdot]} & 0 & 0 & 0 \\ 0 & \beta_1^{[\cdot]} & \alpha_2^{[\cdot]} & 0 & 0 \\ 0 & 0 & \beta_2^{[\cdot]} & \alpha_3^{[\cdot]} & 0 \\ 0 & 0 & 0 & \beta_3^{[\cdot]} & \gamma^{[\cdot]} \end{bmatrix}$$

and

$$B_{[\cdot]}(t) = \begin{bmatrix} \delta_0^{[\cdot]} & 0 & 0 & 0 & 0 \\ \tau_0^{[\cdot]} & \delta_1^{[\cdot]} & 0 & 0 & 0 \\ 0 & \tau_1^{[\cdot]} & \delta_2^{[\cdot]} & 0 & 0 \\ 0 & 0 & \tau_2^{[\cdot]} & \delta_3^{[\cdot]} & 0 \\ 0 & 0 & 0 & \tau_3^{[\cdot]} & \varepsilon^{[\cdot]} \end{bmatrix}$$

where the placeholder $[\cdot]$ can be X, D, T, S, N. The structures of the matrices $A_{[\cdot]}(t)$ and $B_{[\cdot]}(t)$ arise directly from the state transition model illustrated in Fig 1.

In addition, we let

$$B_{HCC}(t) = b_{HCC}(t)[0 \quad 0 \quad 0 \quad 0 \quad 1]$$
$$B_{HCC-SVR}(t) = b_{HCC-SVR}(t)[0 \quad 0 \quad 0 \quad 0 \quad 1]$$
$$B_u = [q_{chr} \quad 0 \quad 0 \quad 0 \quad 0]'$$
$$B_{DC}(t) = b_{DC}(t)[0 \quad 0 \quad 0 \quad 0 \quad 1]$$
$$B_{DC-SVR}(t) = b_{DC-SVR}(t)[0 \quad 0 \quad 0 \quad 0 \quad 1]$$

The above-defined quantities are then combined into the following matrices, which are used in the state-space model equations (10), (11):

$$A(t) = \begin{bmatrix} A_X & & & & & & & & \\ B_D & A_D & & & & & & & \\ & B_T & A_T & & & & & & \\ & & B_S & A_S & & & & & \\ & & B_N & & A_N & & & & \\ B_{DC} & B_{DC} & B_{DC} & B_{DC-SVR} & B_{DC} & A_{DC} & & & \\ B_{HCC} & B_{HCC} & B_{HCC} & B_{HCC-SVR} & B_{HCC} & & A_{HCC} & & \\ & & & & & B_{LT-DC} & B_{LT-HCC} & & \\ & & & & & & & B_{PT} & A_{PT} \end{bmatrix}$$

$$B(t) = [(1-d_a)B_u' \quad d_aB_u' \quad \mathbf{0_5} \quad \mathbf{0_5} \quad \mathbf{0_5} \quad 0 \quad 0 \quad 0 \quad 0]'$$

$$C(t) = \begin{bmatrix} \frac{B_{HCC}}{c_{HCC}} & \frac{B_{HCC}}{c_{HCC}} & \mathbf{0_5} & \frac{B_{HCC-SVR}}{c_{HCC}} & \frac{B_{HCC}}{c_{HCC}} & 0 & 0 & 0 & 0 \\ C_{CHC} & \mathbf{0_5} & \mathbf{0_5} & \mathbf{0_5} & \mathbf{0_5} & 0 & 0 & 0 & 0 \end{bmatrix}$$

$$D(t) = [0 \quad d_a]'$$

Where c_{HCC} is given in S3 Table, $C_{CHC} = [d_0 \quad d_1 \quad d_2 \quad d_3 \quad d_4 + d_{HCC} + d_{DC}]$ and $\mathbf{0_5}$ denotes a row vector of five zeros.
