# Supplementary material for: A model-based framework for chronic hepatitis C prevalence estimation
Source: PLoS One. 2019 Nov 21;14(11):e0225366. doi: 10.1371/journal.pone.0225366 (PMC6874092; doi:10.1371/journal.pone.0225366)
Supplement: S2 Appendix — (PDF) [file pone.0225366.s008.pdf]

S2 Appendix - Construction of the model likelihood function

The data we used in model calibration can be categorized into: time-series data $z_{ts}(t) = [z_{HCC}(t) \quad z_{hepC}(t)]'$, (for $t = 0,\cdots,14$) and liver fibrosis distribution data $z_F = [z_{F_0},\cdots,z_{F_4}]'$. We let $\boldsymbol{z} = [z_{ts}(0)',\cdots,z_{ts}(14)',z_F{}']'$.

The likelihood function $L(M|\boldsymbol{z},V)$ is the probability of observing the data vector $\boldsymbol{z}$ given the parameter vector of unknown parameters M and uncertain parameters V. This likelihood function can be decomposed into a product of likelihood functions for the time series data ($L_{ts}(M|z(0),\cdots,z(14),V)$) and for the fibrosis distribution data ($L_F(M|z_{F_0},\cdots,z_{F_4},V)$). Formally:

$$L(M|\boldsymbol{z},V) = L_F(M|z_{F_0},\cdots,z_{F_4},V)L_{ts}(M|z_{ts}(0),\cdots,z_{ts}(14),V)$$

To construct L_{ts}, we make use of the definitions of the observables in (6) and (9). We assume that, for each t, $z_{HCC}(t)$ follows a Poisson distribution with mean $y_{HCC}(t)$ and $z_{hepC}(t)$ follows another Poisson distribution with mean $y_{hepC}(t)$. The number of HCC diagnoses $z_{HCC}(t)$ conditional upon $y_{HCC}(t)$ can be assumed to be independent of the number of diagnoses $z_{hepC}(t)$ conditional upon $y_{hepC}(t)$. For the time series data, the likelihood function is

$$L_{ts}(M|z(0),\cdots,z(14),V) = \prod_{t=0}^{14}\left(L_{HCC}(y_{HCC}(t)|z_{HCC}(t),V)\cdot L_{hepC}(y_{hepC}(t)|z_{hepC}(t),V)\right)$$

where, for $j = HCC,\ hepC$, $L_j(y_j(t)|z_j(t),V) = \frac{y_j(t)^{z_j(t)}}{z_j(t)!}\exp(-y_j(t))$.

To construct L_F, we use the definitions of the observables in (7) and (8). Let $Y_{CHC} = \sum_{t=0}^{14} y_{CHC}(t)$ and let $Y_{F_i} = \sum_{t=0}^{14} y_{F_i}(t)$ for $i = 0,1,2,3,4$. Given Y_{CHC} and z_{F_i} from S5 Table, we expect that, on average, $z_{F_i}Y_{CHC}$ (±25%) CHC diagnoses at fibrosis level F_i over the years $t = 0,\cdots,14$. The model, conditioned on M and V, estimates Y_{F_i} CHC diagnoses at fibrosis level F_i over the years $t = 0,\cdots,14$. We model Y_{F_i} as being normally distributed around a mean of $z_{F_i}Y_{CHC}$ with variance $\sigma_{F_i}^2$. Taking the ±25% error in the mean

as being equal to two standard deviations, we set $\sigma_{F_i} = \frac{1}{8} z_{F_i} Y_{CHC}$. The contribution to the model likelihood arising from fibrosis distribution data therefore satisfies

$$L_F\left(M \middle| z_{F_0}, \cdots, z_{F_4}, V\right) \propto \prod_{i=0}^{4} \exp\left(-\left(\frac{Y_{F_i} - z_{F_i} Y_{CHC}}{2\sigma_{F_i}^2}\right)^2\right).$$
