# Supplementary material for: A model-based framework for chronic hepatitis C prevalence estimation
Source: PLoS One. 2019 Nov 21;14(11):e0225366. doi: 10.1371/journal.pone.0225366 (PMC6874092; doi:10.1371/journal.pone.0225366)
Supplement: S3 Appendix — (PDF) [file pone.0225366.s009.pdf]

S3 Appendix - Details of model fitting algorithm.

Algorithm 1: Algorithm to obtain a distribution of calibration parameter sets M for a single birth cohort.

Input: Equations (12) and (13).
Parameter distributions $P(V)$.
Calibration parameter prior distributions $P(M)$.
Calibration data in S4 Table and S5 Table.

Output: Collection of parameter sets $\mathcal{S}$, sampled from $P(M, V|\mathbf{z})$.

Initialize $K_1 \leftarrow 10^4$, $K_2 \leftarrow 10^6$, $K_3 \leftarrow 10^6$
// Stage 1 – Obtain posteriors conditioned on V
for $k = 1$ **to** $k = K_1$**:**
  Initialize $c_k \leftarrow 1$
  $V_k \leftarrow$ Sample from $P(V)$
  $M_{k,1} \leftarrow$ Sample from $P(M)$
  for $j = 2$ **to** $j = K_2$**:** //Start Metropolis-Hastings
    $M_j \leftarrow$ Sample from proposal distribution $\rho(M_j|M_{k,c_k})$
    $r \leftarrow$ Sample from Uniform(0,1)
    if $a(M_j|M_{k,c_k}) \geq r$**:** //M_j acceptance test (S-1, S3 Appendix)
      $c_k \leftarrow c_k + 1$ //Update no. accepted parameter sets
      $M_{k,c_k} \leftarrow M_j$ //Assign accepted sample to M_{k,c_k}
// Stage 2 – Marginalization over V
Initialize $\mathcal{S} \leftarrow \emptyset$
for $k = 1$ **to** $k = K_3$**:**
  $V_{k^*} \leftarrow$ Sample $\{V_1, \cdots, V_{K_1}\}$ //Sample V_{k^*} from $P(V)$
  $M_{k^*,c^*} \leftarrow$ Sample $\{M_{k^*,c_1}, \cdots, M_{k^*,c_k}\}$ //Sample M_{k^*,c^*} from $P(M|\mathbf{z}, V_{k^*})$
  $\mathcal{S} \leftarrow \mathcal{S} \cup [V_{k^*}, M_{k^*,c^*}]'$

The Metropolis-Hastings algorithm [7] is implemented in the inner for loop of Algorithm 1. The algorithm is initiated with a vector of calibration parameters $M = M_1 = [m_{1_1} \quad \cdots \quad m_{1_J}]'$ that is obtained by sampling the prior distributions in Table 1. Next, in the inner loop, the algorithm samples a new vector of calibration parameters M_2 from Table 1 conditional upon M_1. The probability distribution $\rho(M_2|M_1)$ used to propose M_2 when the initial model is M_1 is given by

$$\rho(M_2|M_1) = \prod_{j=1}^{J} \rho_j\left(m_{2_j}|m_{1_j}\right)$$

where $\rho_j\left(m_{2_j}|m_{1_j}\right)$ is a normal distribution with a domain limited to the parameter's allowable values.

Once model M_2 is proposed, the algorithm accepts it as the next step in the Markov chain with probability $a(M_2|M_1)$. From [7], this acceptance probability is given by

$$a(M_2|M_1) \coloneqq \min\left(1, \frac{\rho(M_1|M_2)P(M_2|\boldsymbol{z}, V_k)}{\rho(M_2|M_1)P(M_1|\boldsymbol{z}, V_k)}\right) \qquad \text{S-1}$$

If the model M_2 is accepted, it is appended to the collection of accepted models under the condition $V = V_k$. Otherwise M_2 is discarded. A new iteration of the inner loop of Algorithm 1 then begins.
