# Supplementary material for: A model-based framework for chronic hepatitis C prevalence estimation
Source: PLoS One. 2019 Nov 21;14(11):e0225366. doi: 10.1371/journal.pone.0225366 (PMC6874092; doi:10.1371/journal.pone.0225366)
Supplement: S4 Appendix — (PDF) [file pone.0225366.s010.pdf]

S4 Appendix - Mean estimates of total numbers of CHC cases and undiagnosed CHC cases.

Estimated mean total number of CHC cases in Canada, 1999-2013, by birth cohort			
**Year**	**Birth years < 1945**	**1945 ≤ Birth years ≤ 1964**	**Birth years > 1964**
**1999**	54341	145367	31916
**2000**	50057	139972	34196
**2001**	48098	138876	37949
**2002**	46123	137389	41797
**2003**	44120	135555	45727
**2004**	42127	133401	49739
**2005**	40164	130965	53834
**2006**	38192	128276	58085
**2007**	36236	125374	62420
**2008**	34311	122288	66832
**2009**	32434	119051	71327
**2010**	30626	115689	75898
**2011**	28854	112234	80642
**2012**	27134	108712	85459
**2013**	25472	105143	90337

Estimated mean total number of undiagnosed CHC cases in Canada, 1999-2013, by birth cohort			
**Year**	**Birth years < 1945**	**1945 ≤ Birth years ≤ 1964**	**Birth years > 1964**
**1999**	34988	118845	24310
**2000**	31003	107681	22494
**2001**	27526	97345	21355
**2002**	24464	87676	20677
**2003**	21752	78646	20365
**2004**	19360	70233	20358
**2005**	17251	62423	20606
**2006**	15369	55200	21097
**2007**	13694	48551	21773
**2008**	12202	42461	22610
**2009**	10875	36917	23593
**2010**	9701	31901	24708
**2011**	8650	27395	25971
**2012**	7711	23376	27345
**2013**	6875	19818	28824
