# Supplementary material for: A model-based framework for chronic hepatitis C prevalence estimation
Source: PLoS One. 2019 Nov 21;14(11):e0225366. doi: 10.1371/journal.pone.0225366 (PMC6874092; doi:10.1371/journal.pone.0225366)
Supplement: S5 Appendix — (PDF) [file pone.0225366.s011.pdf]

S5 Appendix - Prior and posterior distributions of year 2013 number of new infections and probabilities of diagnosis.

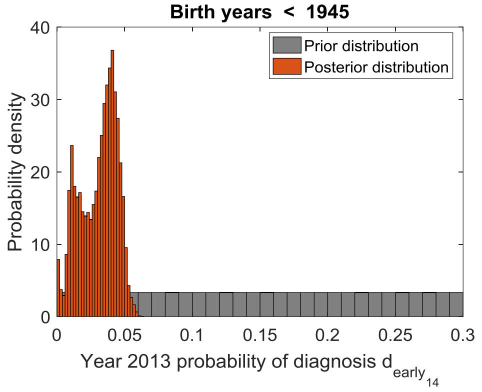

A – Year 2013 prior and posterior distributions of CHC diagnosis probability among patients in fibrosis stages F0 and F1 ($d_{early_{14}}$) born before 1945.

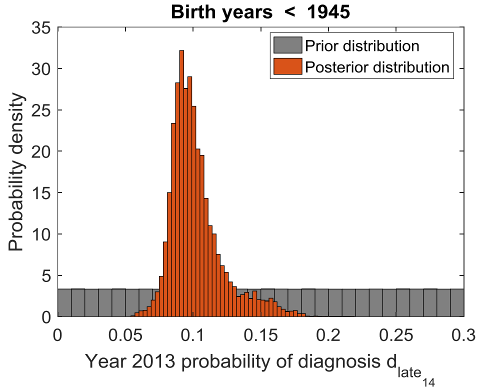

B – Year 2013 prior and posterior distribution of CHC diagnosis probability among patients in fibrosis stages F2-F4 ($d_{late_{14}}$) born before 1945.

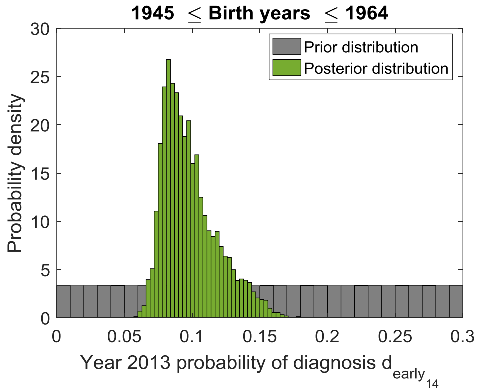

C – Year 2013 prior and posterior distributions of CHC diagnosis probability among patients in fibrosis stages F0 and F1 ($d_{early_{14}}$) born in 1945-1964.

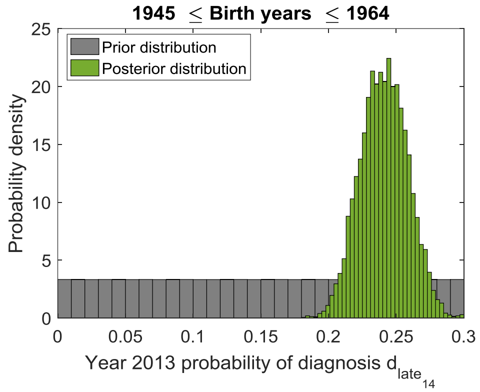

D – Year 2013 prior and posterior distributions of CHC diagnosis probability among patients in fibrosis stages F2-F4 ($d_{late_{14}}$) born in 1945-1964.

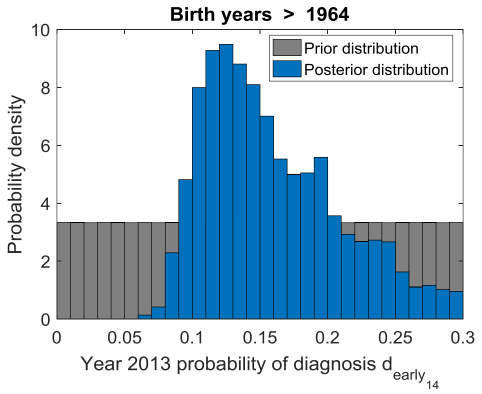

E – Year 2013 prior and posterior distributions of CHC diagnosis probability among patients in fibrosis stages F0 and F1 ($d_{early_{14}}$) born after 1964.

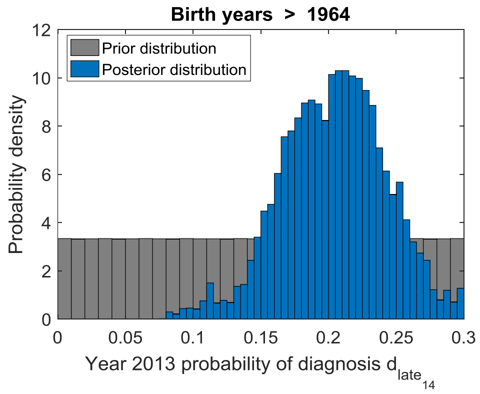

F – Year 2013 prior and posterior distributions of CHC diagnosis probability among patients in fibrosis stages F2-F4 ($d_{late_{14}}$) born after 1964.

S5 Appendix - continued

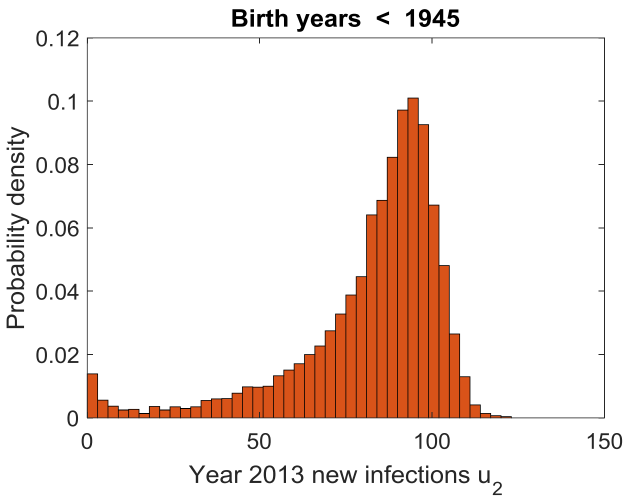

A - Year 2013 posterior distribution of number of new infections among individuals born before 1945. Prior distribution is uniform with range [0, 2×10^4]

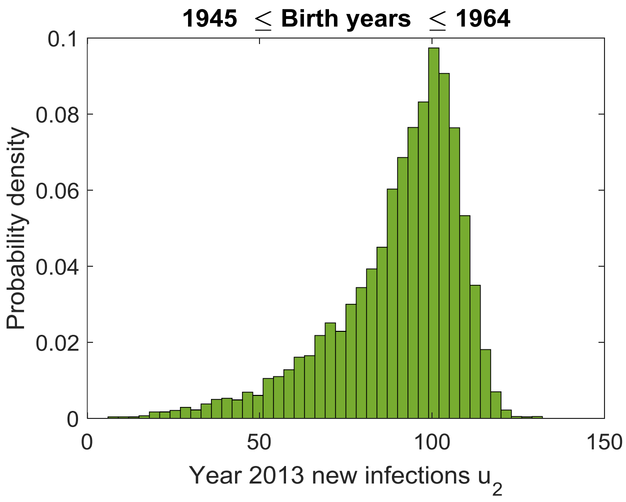

B - Year 2013 posterior distribution of number of new infections among individuals born in 1945-1964. Prior distribution is uniform with range [0, 2×10^4]

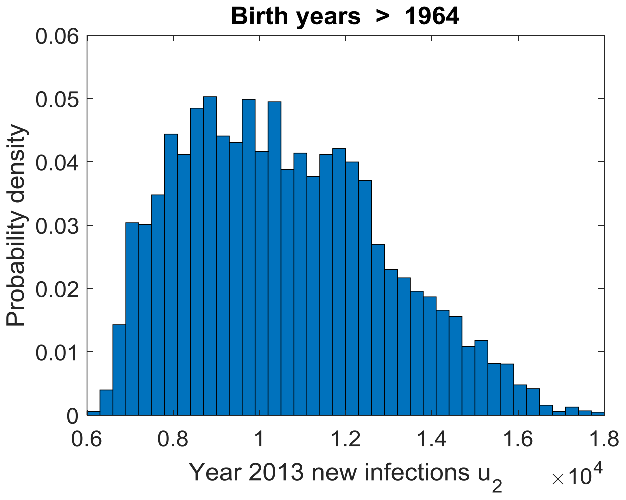

C - Year 2013 posterior distribution of number of new infections among individuals born after 1964. Prior distribution is uniform with range [0,2×10^4]
